# An Ecosystem for Digital Reticular Chemistry

**DOI:** 10.1021/acscentsci.2c01177

**Published:** 2023-03-10

**Authors:** Kevin Maik Jablonka, Andrew S. Rosen, Aditi S. Krishnapriyan, Berend Smit

**Affiliations:** † Laboratory of molecular simulation (LSMO), Institut des Sciences et Ingénierie Chimiques, Ecole Polytechnique Fédérale de Lausanne (EPFL), Rue de l’Industrie 17, CH-1951 Sion, Switzerland; ‡ Department of Materials Science and Engineering, University of California, Berkeley, California 94720, United States; ∇ Miller Institute for Basic Research in Science, University of California, Berkeley, California 94720, United States; § Materials Science Division, Lawrence Berkeley National Laboratory, Berkeley, California 94720, United States; ∥ Department of Chemical and Biomolecular Engineering, University of California, Berkeley, California 94720, United States; ⊥ Department of Electrical Engineering and Computer Science, University of California, Berkeley, California 94720, United States; # Computational Research Division, Lawrence Berkeley National Laboratory, Berkeley, California 94720, United States

## Abstract

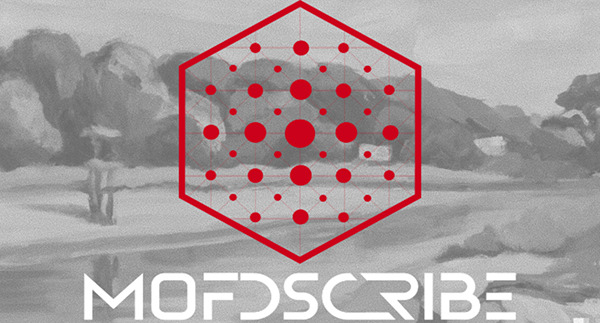

The vastness of the materials design space makes it impractical to explore using traditional brute-force methods, particularly in reticular chemistry. However, machine learning has shown promise in expediting and guiding materials design. Despite numerous successful applications of machine learning to reticular materials, progress in the field has stagnated, possibly because digital chemistry is more an art than a science and its limited accessibility to inexperienced researchers. To address this issue, we present mofdscribe, a software ecosystem tailored to novice and seasoned digital chemists that streamlines the ideation, modeling, and publication process. Though optimized for reticular chemistry, our tools are versatile and can be used in nonreticular materials research. We believe that mofdscribe will enable a more reliable, efficient, and comparable field of digital chemistry.

## Introduction

Reticular chemistry, the science of constructing extended crystalline structures from molecular building blocks, gives scientists a unique playground for material design and discovery as it gives access to a practically infinite-dimensional design space across many length scales: One can architect the pore, functionalize the building blocks, or even encode chemical sequences across unit cells.^1^ These possibilities made reticular chemistry one of the most active fields of modern chemistry with more than 100000 structures collected in experimental databases.^2^ It is not possible to explore this entire design space by mere trial-and-error using brute-force computational screenings and iterative experimental testing. Similar to many other scientific domains,^3−5^ this realization gave rise to the notion of *digital reticular chemistry*,^6^ and in particular to the use of data-intensive research to aid the discovery and design of new reticular materials for any given application by learning predictive models from data.^7,8^ Thus far, machine-learning approaches have—among others—been used to predict gas adsorption properties,^9^ colors,^10^ oxidation states,^11^ electronic properties,^12,13^ heat capacities,^14^ or synthesis conditions^15^ as well as (water) stability of metal–organic frameworks (MOFs).^16−18^

This work is motivated by the observation that the full potential of data science in this field has not yet been achieved. In fact, we argue that some critical bottlenecks limit our progress. Even though all works operate on the same class of materials and often use related machine-learning approaches, these works are hardly comparable or replicable and only implementable by experienced groups. This impediment is present across all stages of the machine-learning workflow. Researchers use different data sets to train and test their models—as we show, sometimes with significant data leakage^19^—preventing direct comparison of modeling approaches. Further down the modeling pipeline, practitioners often use different implementations of the same technique to convert structures into feature vectors—or do not attempt to try different strategies due to implementation challenges. At the end of the modeling process, models need to be validated. However, also there, researchers use different protocols, and—as we discuss—not always the most meaningful ones. Together with the lack of platforms that compile the results obtained with different approaches, these bottlenecks make machine learning for reticular chemistry still more an art than a science.

For many machine learning applications, it has been observed that these problems can be overcome by providing a proper scientific ecosystem for the field: providing the basic building blocks for all the relevant steps in an easily accessible form.^20^ If such a software ecosystem is in place, users can radically accelerate the pace of innovation (as they can use interoperable building blocks and reuse others’ work) while ensuring that their work contributes to the advancement of the field. In this work, we report such a software ecosystem that aims to achieve this goal.

Our ecosystem provides machine learning-ready data sets, along with more than 40 reported and novel featurization approaches, under a consistent application programming interface (API) that enables rapid experimentation and makes those tools accessible to nonexperts. Moreover, to facilitate consistent and meaningful evaluations of machine learning approaches, we also provide data splitters, as well as benchmarking tools that allow submission to a public leaderboard that is automatically updated upon submission.

Using materials design case studies, we illustrate the importance of these best practices; negligence of which can, in some cases, lead to the selection of models with much worse generalization performance.

Importantly, while our tools are optimized to address the challenges and opportunities of reticular chemistry, most, if not all, of them can also be applied to other material classes. This applies also to our case studies, such as the one about the impact of data leakage.

## Results and Discussion

Machine learning studies typically need to go through multiple, often iterative stages, all supported by our mofdscribe software library.

① *Collecting a data set.* For machine learning efforts to be comparable, consistent data sets, along with measures that mitigate data leakage, are needed.
  In mofdscribe, we provide a consistent interface to multiple commonly used data sets^2,21−24^ as well as a completely new data set of adsorption properties, complementing the QMOF database.^12,13^ Additionally, we implement measures to mitigate the effects of data leakage.
② *Featurizing a material.* Most machine learning models only accept inputs of fixed shape. Therefore, structures (which generally have varying numbers of atoms) need to be converted into fixed-sized arrays. However, since for some of these strategies, there are no reusable open-source implementations, or existing ones are hard to combine, researchers seldom explore different featurization approaches.
  To address this, we implemented more than 40 different such featurization strategies that have been used in the literature as well as completely new ones.
③ *Splitting the data sets.* To estimate the generalization performance of a model, it needs to be evaluated on data it has not seen before (i.e., is independent of the training data) and, ideally, mimics the distribution of data the model will be used on.^19,25^ For this, one typically splits the data set collected in step ① into multiple parts. However, as we show, the chosen strategy can have an important impact on model selection and interpretation of the results.
  Therefore, mofdscribe implements multiple reported as well as novel splitting strategies to ensure stringent model evaluation.
④ *Evaluating performance.* Moreover, to compare and evaluate models, we need to compute metrics.^26^ However, as we argue below, practitioners tend to report commonly used metrics instead of ones that are actually relevant for the application.
  We showcase such a more relevant metric and implement it along with others in the mofdscribe package.
⑤ *Comparing the performance with the state-of-the-art.* For science to make progress, it is important to be able to compare with and build on top of others’ results. In the current state of digital reticular chemistry, this is not possible. To address this, mofdscribe implements benchmarking tools that allow direct submission to task-specific leaderboards. Furthermore, the design of our benchmarking tool requires users to also share their hyperparameter optimization strategies.

### Structure Data Sets

Many machine learning practitioners recognize benchmark sets as drivers of progress. For instance, researchers in image classification can easily compare the performance of competing approaches, as they can compare model performance on the same tasks on the same data set (e.g., ImageNet^27^).^28^ Over the last few years, similar benchmark data sets have been reported for generative models for molecules^29^ or quantum machine learning.^30−34^ However, there is currently no widely used reference set for machine learning on metal–organic frameworks (even though the QMOF data set^12,13^ makes important steps toward this goal). As a first step toward more comparable machine learning for MOFs, our package implements a consistent interface for collections of structures, along with some corresponding properties (e.g., gas adsorption or electronic properties) with which all data sets can be used via the same interface. Our package implements reference data sets based on the QMOF database,^12,12^ the ARC database,^22^ the BW database,^21,35,36^ the ARABG database,^37^ as well as on a subset of the CoRE-MOF database.^23,24^

A challenge with the currently existing data sets is that different properties are computed for different structures. However, for many learning applications, it can be useful to have multiple properties for the same structure. To address this, we used reproducible computational workflows^38^ to compute diverse gas separation properties (CO_2_, CH_4_, H_2_, N_2_, O_2_ isotherms; H_2_S, H_2_O, Kr, Xe Henry coefficient as well as parasitic energy for carbon capture from natural gas and a coal-fired power plant^39,40^) for nearly seven thousand materials from the QMOF database (which contains many nonporous materials) and make them accessible via our mofdscribe package. This makes it, to the best of our knowledge, the first database of some gas adsorption properties that are collected alongside many other properties (e.g., bandgap computed with different functionals) of the same structure. We intend to update the database in parallel with the QMOF database.

### Data Leakage

A pitfall for machine learning studies is data leakage, which means information from the test set is leaked into the training.^19^ Often this can happen if, for instance, hyperparameters are tuned based on metrics computed on the holdout test set. However, data leakage can be much more subtle. For example, slight variations of the same structure might occur multiple times in one data set. Machine learning based on data extracted from experimental crystallographic databases [such as the Cambridge Structural Database (CSD)^41^ or the Open Crystallographic Database (COD)]^42^ is particularly prone to this kind of data leakage as one structure can appear multiple times under different identifiers in the database. This can, for instance, be the case because there are different refinements for the same structure or because the measurement was performed at different temperatures. The presence of duplicates in MOF databases has been reported before^43,44^ but is seldom taken into account in machine learning studies. In the Appendix (Case Study 1), we illustrate that for MOFs data leakage is indeed a severe and perhaps underestimated problem.

**Figure 1:**
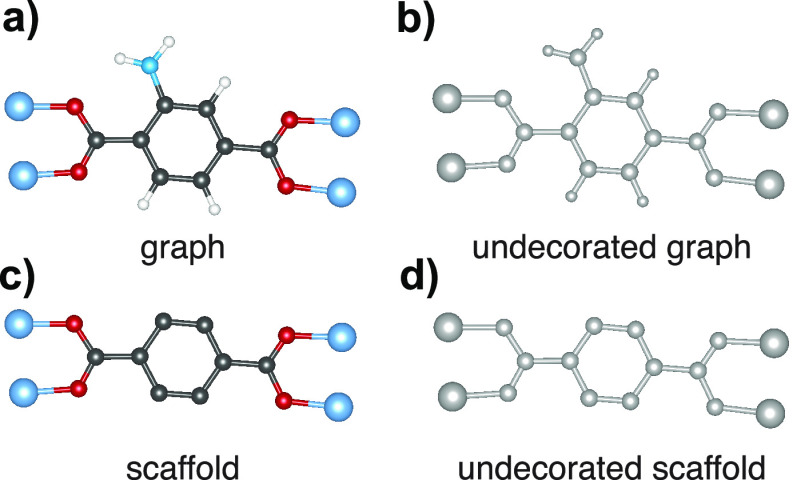
Levels of structure graph abstraction. (a) The (decorated) structure graph considers all atoms, bonds, and atomic numbers as “coloring” of the graph. Therefore, structures with slightly different geometries (e.g., experimental vs DFT-optimized) but the same connectivity will be considered equivalent. (We recently used this definition to find duplicated structures in the Cambridge Crystallographic Database (CSD) when matching structures with their isotherms.^47^) (b) To find structures with the same connectivity but different coloring (e.g., Mg-MOF-74 and Ni-MOF-74), we can use the undecorated graph. (c) A harsher measure of structural similarity can be obtained by only considering the scaffold. Here we form the scaffold by breaking all so-called bridges. Bridges are edges (i.e., bonds) whose breakage leads to an increase in the number of connected components. Practically, those are usually coordinated solvents, hydrogen atoms, functional groups, or other terminal atoms. Note that this definition of scaffold is different from the one that has been used for (Bemis-Murcko) scaffold analysis of molecules.^48^ Therefore, fluorine, chlorine, or amine-functionalized structures would all be treated equally. (d) Also, here, we can remove the coloring to make, for instance, Ni-MOF-74-NH_2_ equivalent to Mg-MOF-74-NH_2_. To simplify the identification of duplicates, we use the Weisfeiler-Lehmann test to convert graphs into a hash string. While this test does not guarantee isomorphism, we found the resulting computational advances drastically outweigh the lack of theoretical guarantees.

To address this problem, mofdscribe implements computationally efficient heuristics that help with the deduplication of data sets. Those heuristics are based on the computation of hash strings of the periodic structure graph (so-called quotient graph),^45^ which describes the connectivity of the atoms in the crystal (vertices being the atom positions and edges being the bonds). Since the structure graph does not directly depend on the exact atomic positions, structures with slightly different atomic positions (e.g., conformers) will share the same structure graph. While a check for graph-isomorphism would be the formally exact way to check for duplicated structure graphs, this can be computationally intensive, or even prohibitive, for large structure graphs as they are common for MOFs. Therefore, we use Weisfeiller-Lehman^46^ hashes of different versions of the structure graphs (Figure 1), corresponding to increasingly tight definitions of duplication. We want to emphasize that while this deduplication strategy is a good default for most applications, it might be too strict for others as, for instance, open and closed forms of a framework will be counted as duplicates. Hence, this automatic deduplication can be disabled and customized in mofdscribe.

### Featurizing Reticular Materials

Before using the data sets to train a model, one typically needs to convert the structures into fixed-length feature vectors. This is required because most machine learning algorithms can only operate on fixed-sized inputs. For instance, we can envision that we want to predict the gravimetric gas uptake of a MOF—a property that should be the same regardless of whether we create a supercell, translate, or rotate the unit cell. That is, we need a function with which the cells of different sizes, or ordering of atoms, are mapped to the same feature vector. This example already illustrates that such a conversion of a structure into a feature vector is not unique and is always connected to certain, often hidden, assumptions. Ideally, those assumptions reflect a physical or chemical understanding of the system and act as so-called inductive biases that help the learning algorithm.^49^ But any approximation always limits the expressivity of the model, and many featurization approaches neglect—by design—certain aspects of a given system. Additionally, there are always certain design choices (such as the numbers that are used to encode chemical elements) that are not ideal for all applications. In mofdscribe we propagate those approximations (such as elemental encodings or aggregations) to the user and therefore also allow tuning those parameters to increase predictive performance.

**Figure 2:**
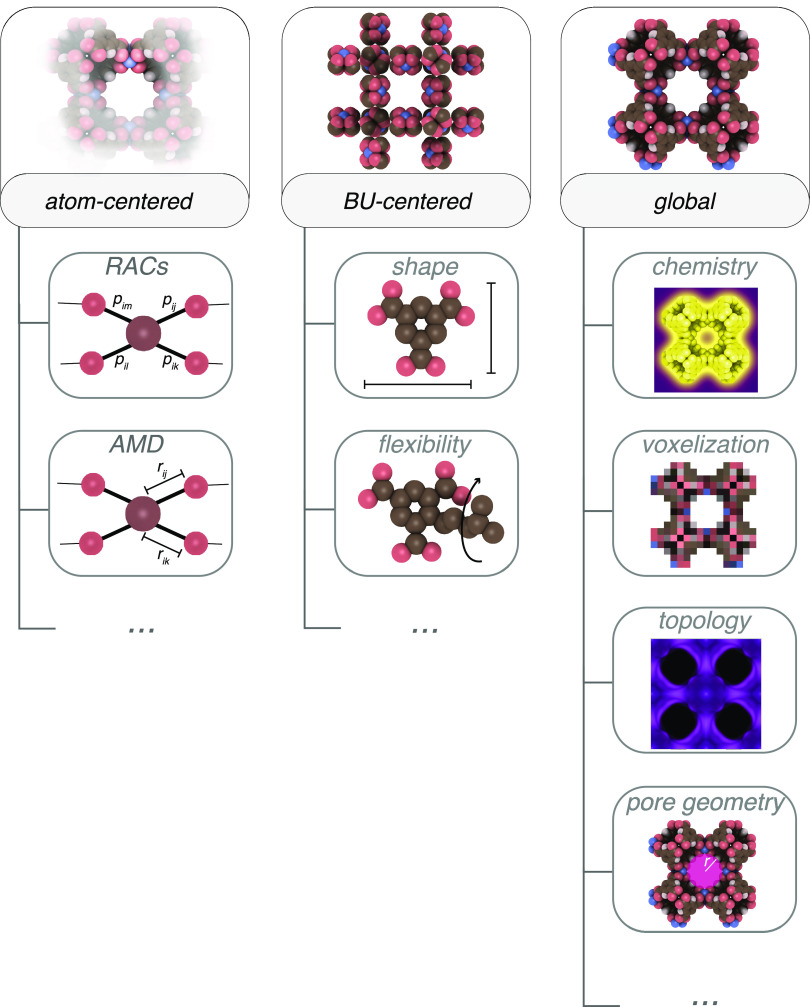
Overview of featurizer types implemented in mofdscribe. We distinguish three scopes on which featurizers operate: atom-centered, building unit (BU-)centered, and global features. Note that mofdscribe is interoperable with matminer, wherefore featurizers implemented in matminer can be used with those implemented in mofdscribe.^50^ For a full overview of implemented featurizers, see Overview of Implemented Featurizers list.

**Scheme 1:**
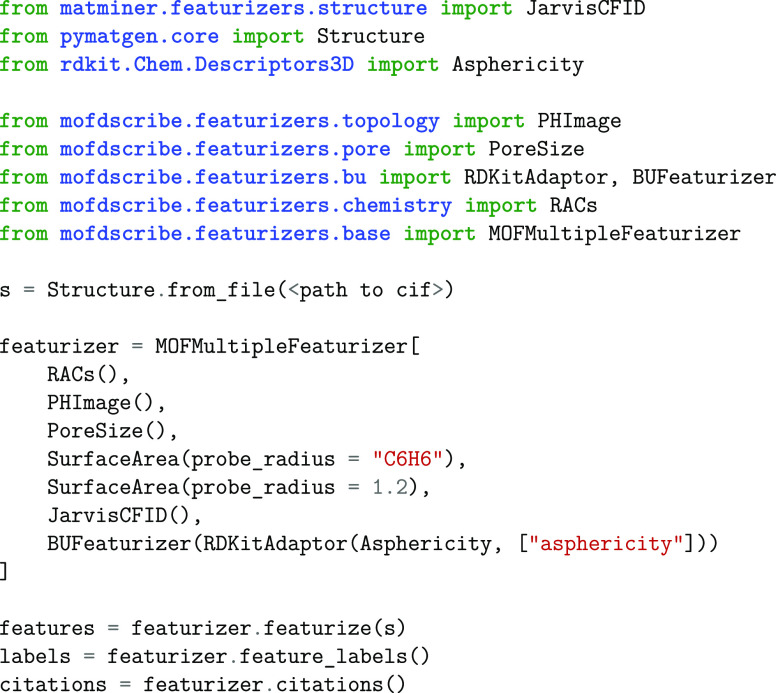
Complete Featurization Usage Example The featurizers in mofdscribe can be easily combined with the ones implemented in matminer. All featurizers also share the same utility methods, such as citation and feature_labels, and can be computed for multiple structures using featurize_many.

A key design aspect for featurizers is the length scale they operate on (Figure 2). In mofdscribe, we distinguish featurizers operating on the local, atom-centered neighborhoods, the building units (BU), and the full, global structure. Depending on the learning tasks, different scales will be more relevant. For instance, for gas separations, we need to describe the textural properties of the pore (global) along with the chemistry of the building unit (BU/atom-centered). Therefore, it is important that featurizers operating on different scales can easily be combined. In mofdscribe, all featurizers can be used in the same way and combined as needed, thereby enabling rapid experimentation. To make this possible, mofdscribe uses the featurizer design pattern popularized by the matminer package (see Scheme 1 in which we compute a feature vector for a MOF by combining featurizers from all scopes). This design pattern, which bears similarities to the sklearn API,^51^ ensures consistency across how different featurizers are used and, in this way, enables composability and also makes them accessible to nonexperts. By building on top of the matminer building blocks, mofdscribe is also fully interoperable with the featurizers implemented in the matminer library. For instance, featurizers such as the matminer’s SiteStatsFingerprint can be seamlessly used to separately featurize framework and guest molecules using the HostGuestFeaturizer implemented in mofdscribe.

#### Locality Approximation

**Scheme 2:**
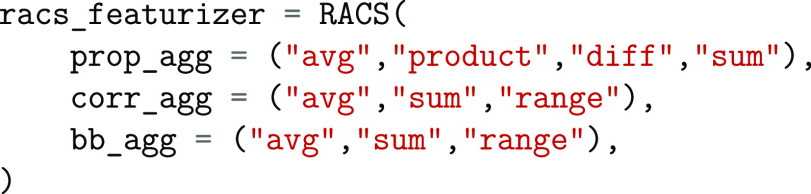
Example of Using Aggregations in mofdscribe Many featurizers compute more than one feature vector per structure, for instance, one feature vector per atom. In this case, the data must be further processed to construct one fixed-size feature vector per structure. To ensure that the resulting feature vectors are permutation invariant (that is, do not depend on the arbitrary numbering of atoms in the structure), one typically uses aggregation functions such as the average, sum, maximum, or minimum. At every point where aggregations are computed, we allow users to customize the ones used. Users can simply specify the desired ones as a tuple of strings; for example, (”median”, ”range”, ”geom_av”) would aggregate the features using the median, range, and geometric average.

In machine learning for chemistry and materials, the most commonly used assumption is the locality approximation. In practice, this assumes that a property does not depend on the entire crystal but that the main contributions are from the local environment (which can be justified based on the principle of “nearsightness of electronic matter”^52^). For example, in our model for the oxidation state of the metal in a MOF,^11^ the features are computed for the metal and the atoms of the linkers surrounding the metal. By reducing the learning problem to local environments, the locality approximation allows a model trained on small fragments to generalize to large structures (which are harder to sample as there are combinatorially more of them).^53^ For reticular materials, this approximation is widely used as part of the feature set (which is often supplemented with global features, see below) via revised autocorrelation functions (RACs),^21,54^ smooth overlap of atomic positions (SOAP) fingerprints,^55^ local geometry descriptors,^11,56^ or the average mean distance descriptor by Widdowson et al.^57^ For all those atom-centered descriptors, one can compute *N* descriptors for a structure with *N* atoms. Since different materials will have different numbers of atoms *N* in their unit cells, one typically needs to perform an aggregation operation, such as computing the arithmetic mean of all the atom-centered feature vectors, to construct a fixed-length descriptor that is permutation invariant. The latter is important since we do not want our descriptors to change when we change the (arbitrary) numbering of atoms (that is only an artifact of digitally encoding materials). Of course, there is not one ideal choice for the aggregation operation. One might benefit from additional expressivity by using multiple aggregations, for instance, the standard deviation alongside the arithmetic mean or other Pythagorean means or robust measures (e.g., trimean, mean absolute deviation). Therefore, in our library, the user can—where applicable—simply provide all aggregation combinations of interest (see Scheme 2 for an example) and mofdscribe will compute them all. As shown in the Supporting Information, this generalization of established descriptors (such as the AMD proposed by Widdowson et al.^57^) can lead to large (>20%) improvements in predictive performance on material property prediction tasks. By exposing all these options, mofdscribe makes these approximations visible to the users and allows users to tune them for better predictive performance.

**Figure 3:**
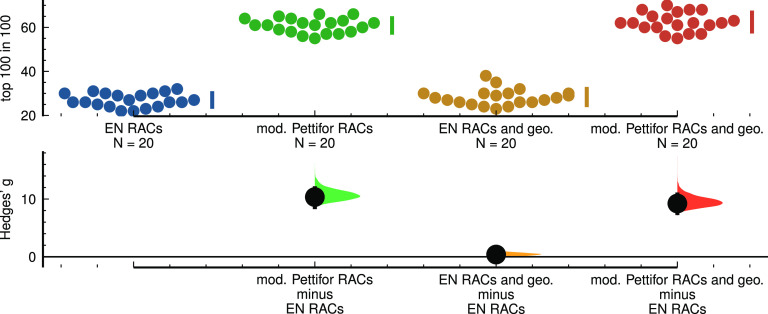
Impact of element encodings on predictive performance. Here, we compare revised autocorrelation functions with different element encodings as input for a gradient-boosted decision tree. We train the model on CoRE-MOF data reported by Moosavi et al.^21^ to predict the logarithm of the CO_2_ Henry coefficient. The top row shows the number of top-100 materials we retrieve in the top-100 predictions of 20 independent runs. The bottom row shows estimated Hedge’s g effect sizes (a suitable effect size metric in the case of little data^61^ which can be thought of as a normalized mean difference) with respect to the performance of the RACs using electronegativity (EN) as element encoding. This figure also compares the performance of feature sets augmented with scalar geometric properties (pore diameters, accessible surface area, and void fraction). We find similar very large trends using other metrics.

Since models do not know the periodic table, the nature of the element types in a given structure needs to be encoded numerically. Often, as in the case of RACs, this is done using element properties such as atomic number or electronegativity. However, it is well-known that some encodings such as atomic numbers lose the clustering of elements according to their periodic properties, which can be an important inductive bias for a machine-learning model. Therefore, mofdscribe allows users to flexibly choose from a wide variety of elemental properties in addition to other encodings such as the (modified) Pettifor scales that have been shown to better capture similarities of elements across the periodic table.^58−60^ For instance, Pettifor scales can be thought of as the “optimal one-dimensional periodic table”:^60^ similar elements are neighboring in this representation (in the original scale with the goal to achieve an optimal map of the stability of AB compounds). The impact the choice of encoding can have is shown in Figure 3, where we find improvements of over 50% by using the modified Pettifor scale^60^ for encoding elements in revised autocorrelation functions (in contrast to using the electronegativity). Also here, mofdscribe exposes these encoding options on all featurizers where they apply. This makes this approximation visible and allows users to tune the encodings to increase the predictive performance or interpretability of their models.

#### Building Unit Centered Descriptors

The defining feature of reticular chemistry is the tinker-toy principle, i.e., the construction of extensive crystal structures from small molecular building blocks. Interestingly, this principle is seldom exploited in machine learning studies for reticular materials such as MOFs. One possible explanation for this is that it is not trivial to extract the building blocks from a crystal structure into a form such that they can be used with featurizers that are conventionally used for molecules (e.g., as the ones implemented in the RDKit program^62^). To facilitate the use of BU-centered featurization we implement an adaptor that can convert any featurization function that accepts a molecule object from the RDKit library (that can easily be generated from a SMILES string) into one that can be used along with all other mofdscribe featurizers. Importantly, to allow the decomposition of MOFs into their building blocks, we also release a library, called moffragmentor, that analyses the structure graph to decompose MOF structures into their building blocks (i.e., metal cluster(s) and linker(s) and possible bound/unbound solvent, algorithm described in the Supporting Information). In contrast to existing tools such as mofid(43) and mBUD,^63^moffragmentor makes them accessible from an object-oriented interface. If a user provides a MOF structure into a featurizer for molecules (e.g., a conformer counter), mofdscribe by default fragments the MOF into building blocks and computes the features separately for each building block. The importance of this step is that once a MOF is decomposed into building blocks, we can also generate descriptors that further characterize these building blocks, for example, descriptors related to the flexibility of the linker such as the number of accessible conformers.^64^ This is an example of a descriptor that could not be easily accessed otherwise but might help digital reticular chemists address questions (e.g., about crystallization) that they could not easily address before. Importantly, all featurizers, e.g., also the SOAP fingerprint, can be used in this setting to compute more meaningful aggregations (in contrast to averages over the full structure). In this process, we do not enforce the use of our moffragmentor library as users can bypass this step by providing their own building blocks. For example, users can provide pymatgen(65)Molecule objects that they obtain by deconstructing MOFs with other tools such as mofid(43) or mBUD.^63^

#### Global Descriptors

In porous materials, many properties—such as the pore size and shape or overall composition—are not directly correlated to atomic environments or their building blocks. Therefore, local descriptors can only implicitly, via large cutoffs, or not at all, represent such properties.

For this reason, practitioners often use local feature sets (e.g., RACs) along with global ones, most commonly with scalars describing the pore geometry (e.g., pore volumes, surface areas, accessible volumes).^66,67^ However, there are additional vector-valued or count-based descriptors^68,69^ that can be used to describe the pore geometry and might be more expressive than scalar descriptors but are seldom used in machine learning studies for reticular materials.

#### Describing the Shape and Chemistry: Persistent Homology

As an alternative to the aforementioned pore geometry descriptors, the use of topological data analysis has been proposed to capture the shape of materials.^70,71^ Topological data analysis can be used to obtain features that are invariant to a continuous transformation of the material structure. Persistent homology, a branch of topological data analysis, captures all the topological information underlying a given point cloud, such as the geometric coordinates of a material. Given a set of points (i.e., atom positions), we can obtain a so-called filtration (e.g., Vietoris-Rips) by continuously increasing the radius of these points to get a family of nested unions of spheres. Persistent homology then tracks the appearance and disappearance of topological features (such as channels and voids) in this filtration. The radius at which a feature appears is the “birth,” while the radius at which a feature disappears is the “death.” The *persistence* of a topological feature is the difference between birth and death, which acts as a measure of how prominent a given topological feature is. The set of all birth-death points is called a persistence diagram. As reticular materials have many channels and voids, persistent homology provides a holistic approach to capturing these topological features. Persistence diagrams are a multiset of birth-death points in the extended plane, and each material can have a different number of points in its persistence diagram. Since most machine learning models operate on points in a fixed-dimensional Euclidean space, one needs to vectorize these diagrams into fixed-size arrays. One can accomplish this, for instance, by computing persistence images, which can be thought of as smoothed versions of persistence diagrams and have been used before in materials science.^72,73^ A challenge with this representation, however, is that it is often very high-dimensional. This can, due to the curse of dimensionality, lead to learning problems (in particular in low-data regimes). As an alternative, we implemented a vectorization method that approximates persistence diagrams using Gaussian mixture models.^74,75^ This allows for low-dimensional representations that can still provide approximate Wasserstein distances (that are conventionally used to measure distances, i.e., a proxy for the difference between persistence diagrams). Additionally, we also implement a simple vectorization as a 2D histogram.

**Figure 4:**
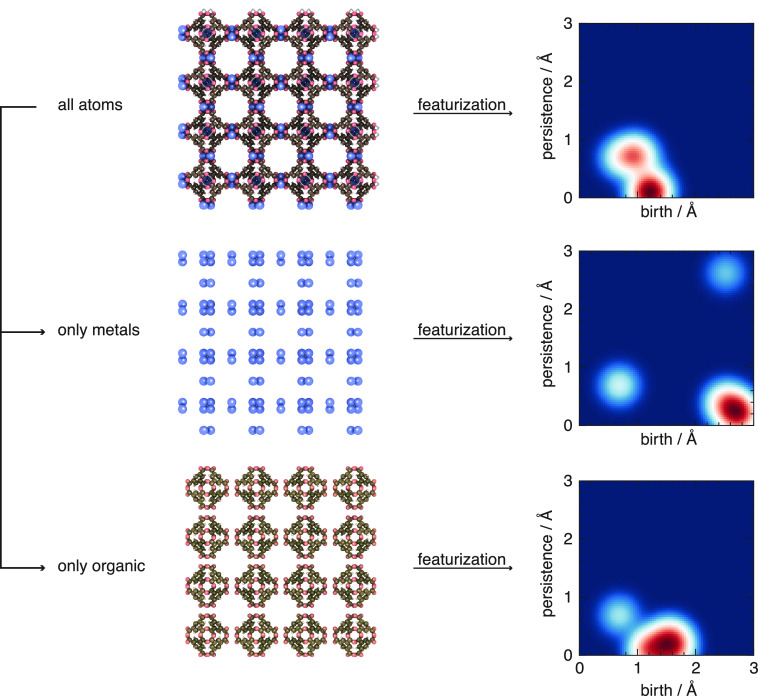
Adding chemical resolution to topological descriptors. Descriptors, such as the ones derived from topological data analysis, operate on the full structure (top row) and capture the geometry and connectivity of a material described by its atomic coordinates. In mofdscribe, we allow users to also incorporate information from different atom types (i.e., not treat all atom types the same way). Users can customize the extent to which they want to lift this many-to-one mapping by adding channels for different atom types. By default, for instance, the descriptors from topological data analysis are computed for all atoms, the metallic substructure, and the organic substructure—all yielding different topological signatures as evident from the persistent images.

**Figure 5:**
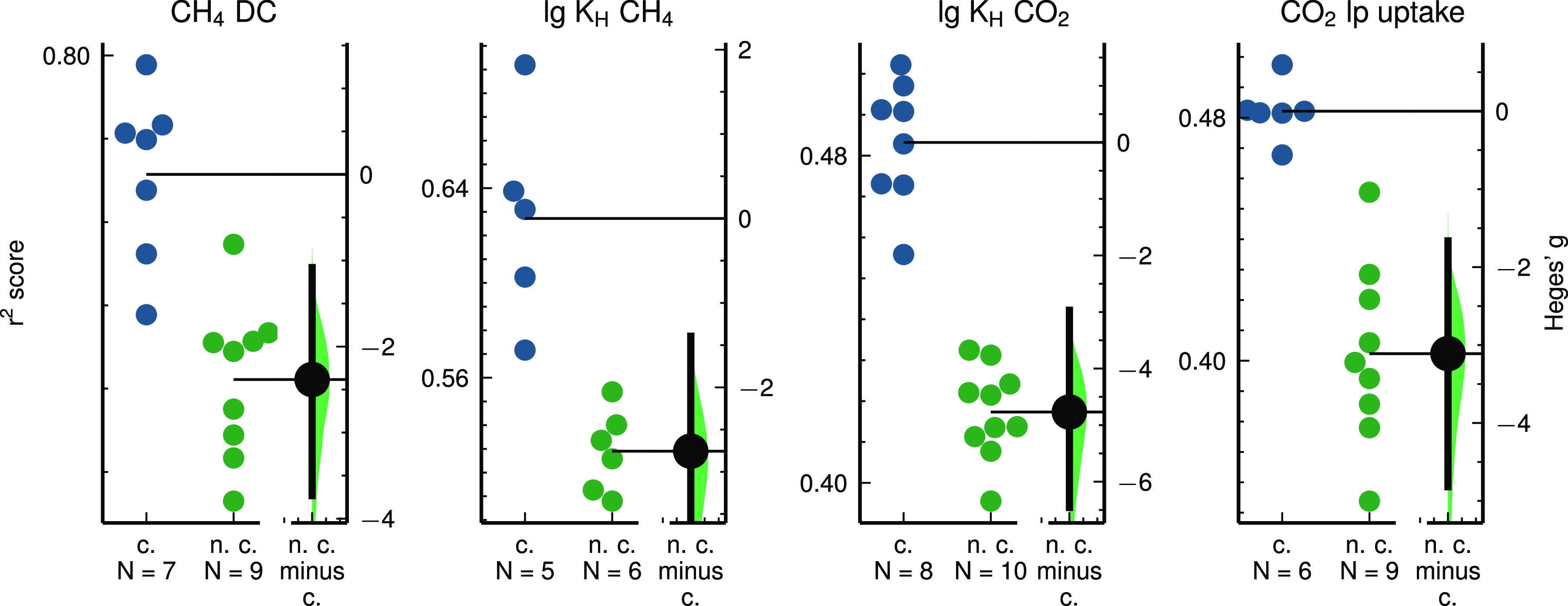
Persistent homology vectors with and without chemical information. In this plot, we use the vectorization of persistent diagrams using Gaussian mixture models with and without chemical information (here, we consider the C–H–N-O, halogen, and metal substructures). For this analysis, we optimize the *full* pipeline (including preprocessing and the model) using automated machine learning.^76^ The plots visualize effect sizes in terms of Hedges’ g. The points indicate the coefficient of determination (*r*^2^ on a holdout test set, shown on the left axes) of the models trained to predict the methane deliverable capacity (DC), Henry coefficient (*K*_H_), as well as the CO_2_ Henry coefficient and low pressure (l.p.) uptake. The blue points are for the model trained with chemistry (c.) information; the green ones indicate the coefficients of determination of the models trained without chemistry information (n.c.). To quantify the effect, we bootstrap the Hedges’ g (a suitable effect size metric in the case of little data,^61^ shown on the right axes) and show it with a kernel density estimate. In all cases, the addition of chemistry shows very large effects.^77^

Topological data analysis captures the geometry of chemical structures and materials, but these systems also have rich chemical information, as they are composed of different atoms. Thus, it is important to incorporate this chemical information into the representation—otherwise, materials with the same connectivity but different elements (for instance, Mg-MOF-74 vs Ni-MOF-74) would be treated the same way, and a model would predict the same properties. To account for chemical information in a highly flexible way, which therefore can adjust to the amount of data available, mofdscribe allows decomposing the structures into structures that contain only certain elements (Figure 4). By default, for instance, mofdscribe will perform the persistent homology analysis on the full structure, the metal substructure, the organic substructure, and the halogen substructure. However, users can customize the substructures that mofdscribe considers and tailor the featurization to the task at hand. As Figure 5 shows, the inclusion of chemical information consistently increases predictive performance on MOF property prediction tasks (in our test cases by up to 20%). We use the same approach to make the average minimum distance fingerprint proposed by Widdowson et al.^57^ chemistry-aware (and observe similar improvements on benchmark tasks there). Additionally, we also allow users to encode chemistry using so-called *weighted* alpha shapes. In this case, in addition to the coordinates, the (atomic) radii of different elements are used for persistence diagram construction to distinguish between different atom types.

#### MOF Tomography

Some of the most successful applications of machine learning have been in computer vision.^78^ The primary reason for this is that while images contain a wealth of information, it is hard to extract good features that can then be fed into a model (e.g., training a linear regressor on a flat vector of all the pixel values will not work). Convolutional neural networks (CNNs) and related architectures are *trainable* feature extractors.^79^ That means, given some data, they can learn to extract the most predictive features (thereby enabling the use of techniques such as transfer learning). However, it is not obvious how one can convert structures, which might have a varying number of atoms and which unit cells might be skewed, into “rectangular” image tensors of fixed size. Additionally, one also needs to consider that one would like to also encode the chemistry of a given material. In mofdscribe, we implement featurizers that voxelize approximately cubic supercells of crystal structures into 3D images (which one could then process using a 3D CNN).^80,81^ Also for these featurizers, we allow the users to use aggregations of custom properties (e.g., Pettifor scale, electronegativities, atomic numbers) as the value for the voxels instead of just binary indication of occupied/unoccupied. Of course, for example, for low-data applications, our approach can also only encode the geometry as a binary encoding, density, or using a truncated distance function. These features will, as initial results in the literature indicate,^80−82^ allow for the use of state-of-the-art computer vision models (including self-supervised pretraining and transfer learning) on reticular materials.

### Consistent Model Evaluation and Benchmarking

Having data sets and standard implementations of featurization algorithms is not all that is needed to make machine learning for reticular materials routine and comparable.^83−85^ To reach standard practices^86^ for digital reticular chemistry, we also need to address model validation and comparison.

#### Estimating Material Discovery Ability

Many machine learning models are built to be useful for materials *discovery*. Discovery implies predicting something *unknown*. However, the common practice of using a random train/test split does not necessarily measure the model performance in predicting the *unknown*. First, a simple random train/test split cannot account for the fact that many databases contain very similar structures (see Appendix (Case Study 2)). As we have shown, (hypothetical) databases often contain multiple structures that are only different in the type or position of one functional group. Dividing such a group of structures with identical scaffolds across train and test sets will not give a faithful measure of the generalization ability of the model as one of the main assumptions of testing is violated—train and test sets are not entirely independent of each other (one might see the functionalized graphs as children of the same scaffold). Second, for practical applications, there will almost always be a data shift; that is, the data distribution the model will be used with will be different from the distribution the model has been trained on. For example, it is well-known that structures in hypothetical databases do not have the same distribution (e.g., lower density, less metal diversity) as structures in experimental databases.^21,87^ This has already been recognized by others such as Meredig et al.^88^ who utilized leave-one-cluster-out cross-validation to estimate extrapolation performance (or Xiong et al.^89^ using *k*-fold forward cross-validation). A similar approach, in which one clusters the principal components of the data into *k* clusters and trains on *k* – 1 clusters and tests on the cluster that was not used for training, is implemented in mofdscribe. As each cluster has specific properties, this method tests how well the model can extrapolate to new properties. If we repeat this procedure for every cluster, we can get an overall measure of the robustness and extrapolation ability.

Additionally, we recognize that one interesting benefit of working with experimental data is that we know when a given structure was first reported; the data is time-stamped.^90^ Inspired by common practice in time-series forecasting, we hence can ask “could we predict the performance of materials discovered after year *X* if we only trained on materials discovered before year *X*?” In particular, we can measure how many of the top *k* materials we can recover in the top *n* predictions by the model. Using mofdscribe, this question can easily be answered.

**Figure 6:**
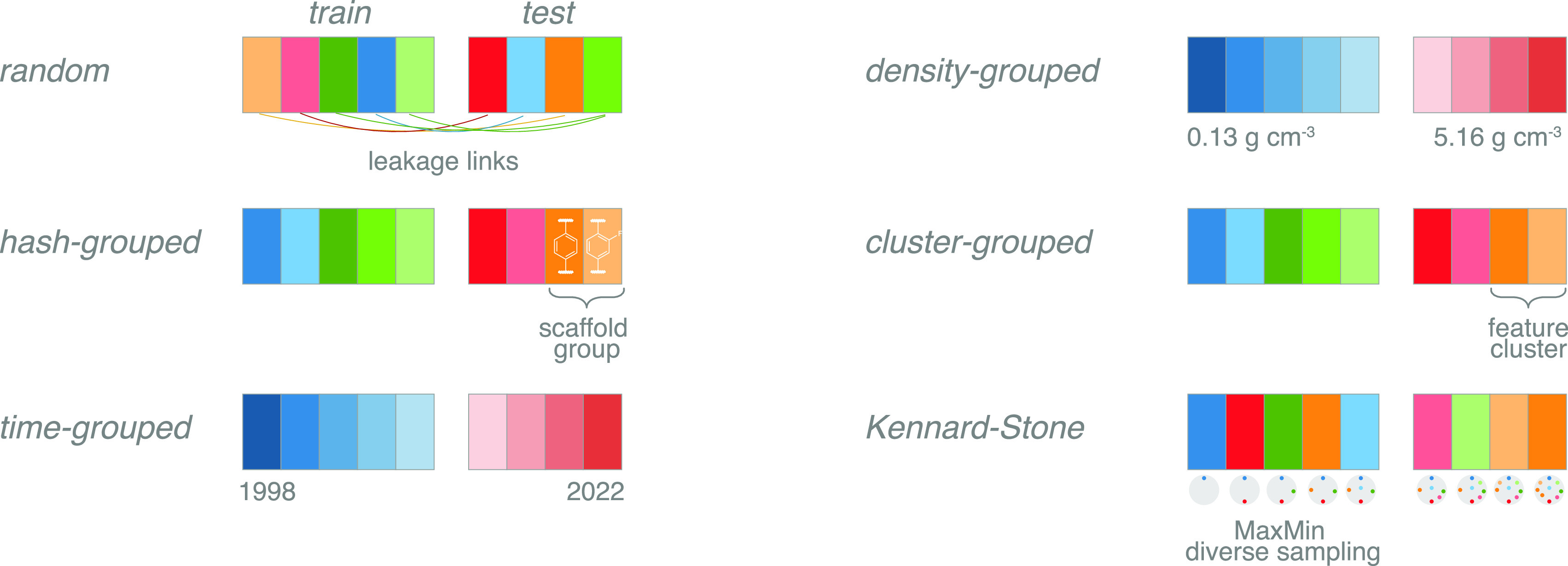
Illustration of some splitting approaches implemented in mofdscribe. For the validation of a machine learning model, it needs to be tested on data it has not seen before. To create sets of such unseen data, data sets are typically split into subsets for training, testing (and validation).^7^ The conventional approach is to perform this split randomly. This, however, might not be a good approximation of real-world use with dependent/grouped data, distribution shift, or lead to problems in the case of imbalanced data. Therefore, mofdscribe implements various splitting strategies that either operate on (structural) features or other metadata. For instance, we extracted the publication date for all structures we could trace back to the CSD and hence allow performing a time-based split. Alternatively, one can use structural features to either ensure equal distribution of the features in different splits (stratification) or to mimic a distribution shift/extrapolation case by forcing different groups into different folds of a cross-validation scheme. The easiest example is to use the density; however, one can also cluster (we use *k*-means clustering after PCA on features, e.g., computed using mofdscribe). Moreover, we also implement a splitting strategy inspired by the scaffold splits sometimes used for molecules^91^ for reticular molecules via our hash strategies (Figure 1). This allows grouping structures with the same connectivity or backbone into the same fold. A different strategy is using Kennard-Stone sampling,^92^ to ensure that the training set is maximally diverse.

**Scheme 3:**
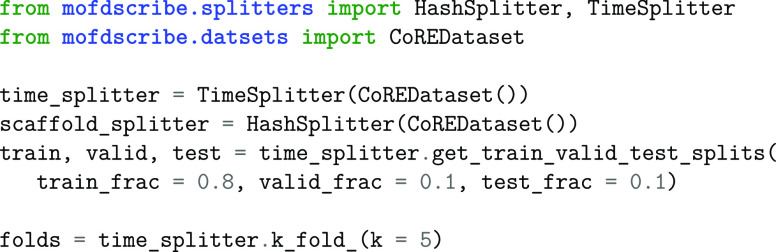
Example of the Use of Splitters The datasets implemented in mofdscribe already provide the relevant information for the splitters (e.g. times, hashes, densities). If the splitters are used on other structures, e.g. custom in-silico assembled MOFs, this information will be computed, if possible on the fly, or can be provided by the user. Note that the datasets, by default, are deduplicated based on the graph hash.

Importantly, a time-based split is not the only feasible splitting strategy—and, depending on the use case, might not be the best option (or might not be applicable if no timestamps are available). Therefore, to further ensure that thorough model evaluation becomes routine for digital reticular chemistry, mofdscribe also implements, inspired by the DeepChem library,^93^ a variety Splitter classes (Figure 6, Scheme 3 for a usage example). The Splitter classes either take a data set that users can define based on their structures or a built-in data set and can produce splits (holdout or *k*-fold cross-validation) following different strategies.

The impact such splitting strategies can have on model selection is shown in the Appendix (Case Study 2), where one can see that the average generalization performance of models significantly depends on the splitting strategy. Overall, we find that the optimal split depends on the task at hand—but typically is not the conventional random split. Given the redundancy in scaffolds in MOF databases, we urge practitioners to use grouped cross-validation.

As a utility to quantify the “difficulty” of the validation strategy, mofdscribe also implements a helper method that performs adversarial validation.^94,95^ Adversarial validation is a technique that has been popularized in data science competitions as a way to measure—with only one number—the difference between training and test distribution but also to identify the most relevant features for a potential difference. For this, one simply trains a classifier to distinguish training from test examples. If the area under the receiver-operating curve (ROC-AUC) is close to 0.5, the classifier fails to distinguish the two data sets. However, if it does not (i.e., ROC-AUC close to 1), analyzing the feature importance can reveal the most relevant features contributing to the difference (which one might decide to remove to improve generalization). An application of this concept is shown in the Appendix (Case Study 2).

#### Benchmarks and Leaderboard

**Scheme 4:**
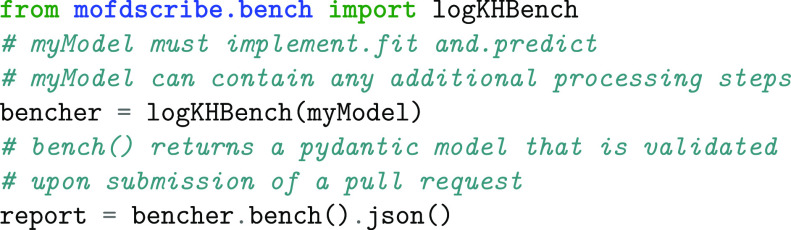
Example of the Use of MOFBench The benchmarking classes only need to be provided with a model object that implements fit and predict methods. It will then use a Splitter object from the mofdscribe package to compute cross-validated metrics on a StructureDataset, which are part of the report. The report also contains additional meta information such as the timings of different steps. It can be serialized to a JavaScript Object Notation (JSON) file that can be submitted to the leaderboard via a pull-request template in the mofdscribe GitHub repository.

To foster the comparability of models built for digital reticular chemistry, we also implemented MOFBench classes that users can use to generate a report of the performance of their modeling pipeline on some benchmark tasks (Scheme 4). The MOFBench classes ensure that all steps are performed consistently and that different modeling strategies become comparable. They define a data set, a splitting strategy, and a set of metrics and automatically capture the computational environment. Via a pull request on GitHub, these results can be easily added to the leaderboard that is currently part of the mofdscribe documentation (mofdscribe.readthedocs.io). Users are additionally asked to provide a file describing the modeling strategy and fill a model card,^19,96^ which will also appear in a subsection of the leaderboard (Appendix (Case Study 3)). We hope that mofdscribe can help pivot reticular chemistry into the digital age by giving the community tools to think and work in a data-driven manner.^97^

## Conclusions

While data-intensive approaches are becoming more popular in (reticular) chemistry, they are still far from being routine and standard; there are currently no standard practices for digital (reticular) chemistry.^86^ We identified the lack of easy-to-use featurization methods and problems with model validation and comparison as the key limitations hampering the progress of the field. To address those impediments, we developed a Python package, mofdscribe, that provides utilities along each step along the path from ideation to model publication. The mofdscribe package provides increased accessibility to machine learning for reticular chemistry and beyond without compromising rigor, especially for less experienced users. This will allow for a closer coupling of data-driven materials design and the synthesis and characterization of (in silico generated) materials since it is very easy for nonexperts to use mofdscribe to power machine-learning models that could be used, for instance, in an active learning workflow.^98^ For this, the adoption of suitable physical workflows will be beneficial. Tools such as the χDL^99^ and the ChemPU^100^ could leverage predictions enabled by mofdscribe to guide an autonomous laboratory if the predictions can be mapped to instructions that can be executed on laboratory robots.^101,102^ While we intend to add new features to the library and maintain it, we also hope to embrace a community effort in which bugs are fixed, data are made more reusable,^103,104^ and new features are added by the community of digital chemists and materials scientists. To facilitate this, we designed our library so that it is easy for researchers to implement new strategies, such as featurizers, in our library so that their work can easily be reused by other digital chemists. We hope that, together with the open availability of machine-actionable data,^105^ our developments will systematize and accelerate machine learning for chemistry.

## Methods

### Featurization

The details of the reimplemented featurizers are described in the original publications and the online documentation.

### Benchmarking using Automated Machine Learning

For the learning curves shown in the Appendix (Case Study 1), we trained gradient-boosted decision trees, as implemented in the XGBoost library, on the default feature set in mofdscribe. To mimic currently utilized settings, we ran the experiments 100 times with a random train/test split of 0.8/0.2.

For the case study analyzing the impact of splitting techniques, we used optimized gradient-boosted decision tree models, as implemented in the CatBoost(106) library, using optuna(107) for a maximum of 100 trials or a timeout of 10 h using the tree of Parzen estimators^108^ sampling strategy. We detail the hyperparameter grid we considered in Supporting Information.

For Figure 5, we followed the approach from the automatminer library^109^ and used automated machine learning, which automatically optimizes over various models and model architectures within a certain computational budget. Concretely, we use the TPOT library,^110−112^ which uses genetic programming for all machine learning pipeline steps, including feature engineering (for instance, using principal component analysis). We used the defaults of 100 generations with a population size of 100 but also limited the search time to 48 h and 5-fold cross-validation.

### Molecular Simulations

All grand-canonical Monte Carlo simulations for the reference data set were performed using the RASPA code,^113^ describing the force-field as a rigid framework with the UFF force field^114^ and a cutoff of 12 Å, whereby we correct for the truncation using analytical tail-corrections.^115^ Simulations were orchestrated using the AiiDA computational infrastructure.^116,117^

## Figures and Tables

**Figure 7:**
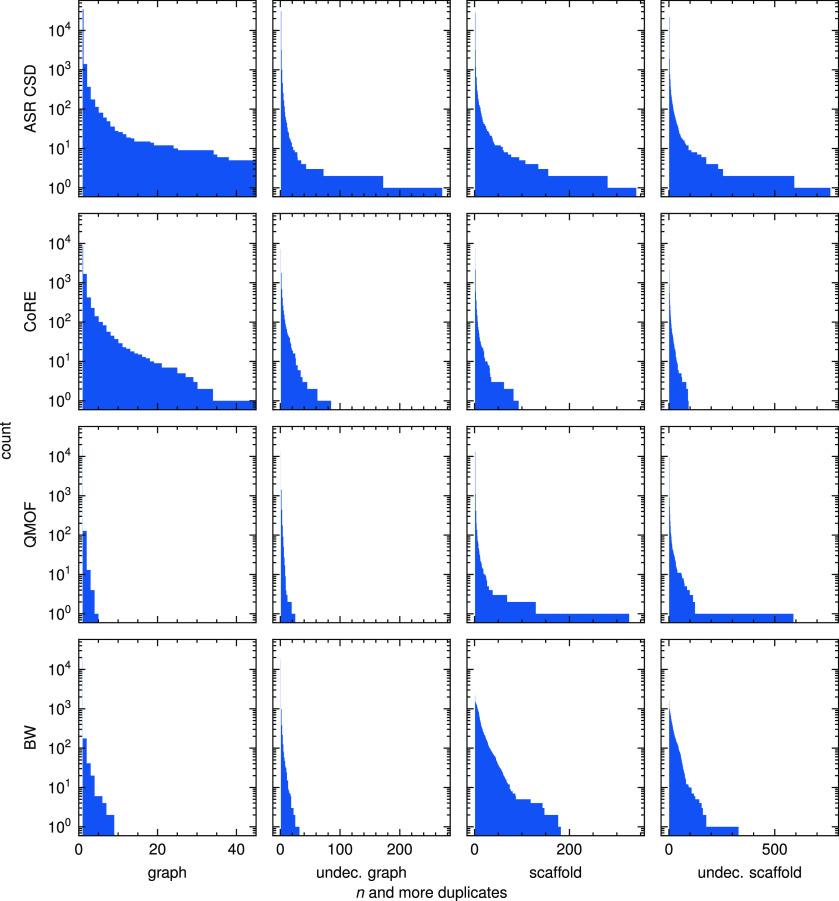
Duplicates across MOF databases. We show the number of duplicates as inverse cumulative histograms; i.e., the bar heights indicate how often we find *n* or more times a duplicated graph type. In the columns, we show increasingly general (i.e., more structures are considered as duplicates) definitions of duplicates. The rows show the counts for different databases: The all-solvent-removed (ASR-CSD) subset of the 2019 MOF subset of the CSD, the CoRE database, the QMOF database, and the Boyd-Woo database (BW) of hypothetical MOFs.

**Figure 8:**
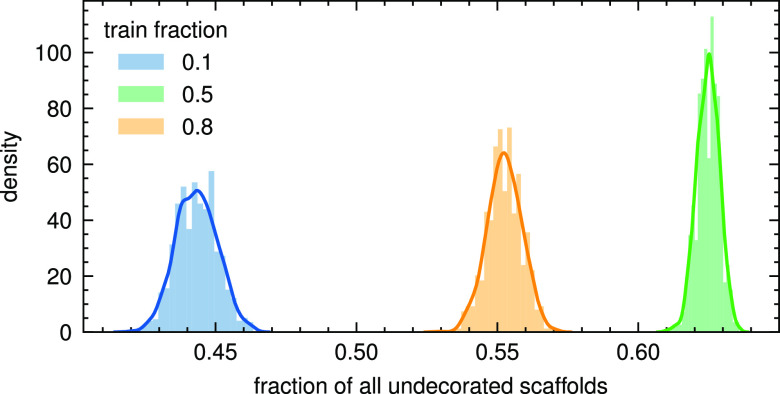
Likelihood of having the same scaffold in train and test set. Using the BW data set implemented in mofdscribe we perform 1000 random train/test split for different train/test ratios and count how often we find an undecorated scaffold hash in both the training and test set. The figure indicates that for commonly used train/test ratios, the majority of scaffolds will be found in both training and test set.

**Figure 9:**
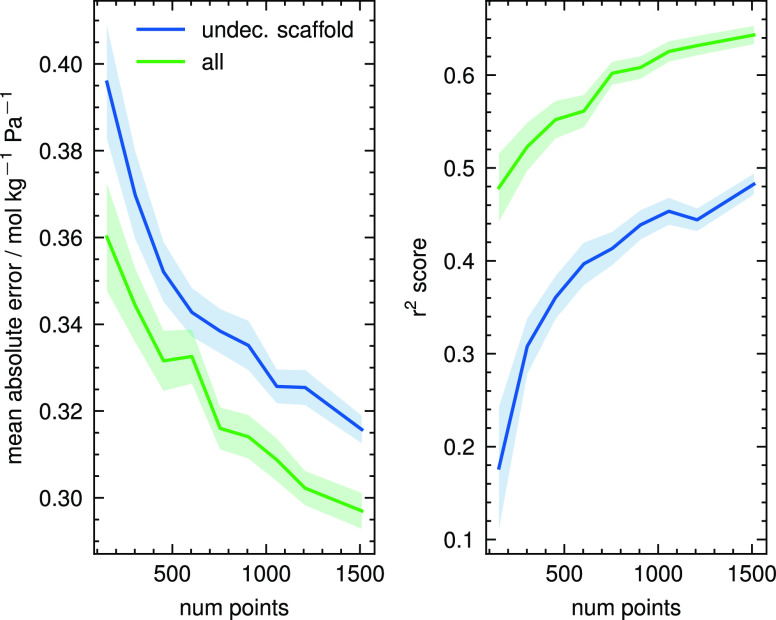
Learning curves with and without duplicated identical scaffolds. For this experiment, we trained gradient-boosted decision trees using the default feature set (currently including histograms of persistence diagrams, AMD, geometric properties, APRDF) implemented in mofdscribe on the BW database subset used in Moosavi et al.^21^ to predict the CO_2_ Henry coefficient (which we reuse from Moosavi et al.^21^). We used a train/test/valid split of 0.7/0.2/0.1 and performed the experiment 100 times. The shaded areas indicate 95% confidence intervals around the mean.

**Figure 10:**
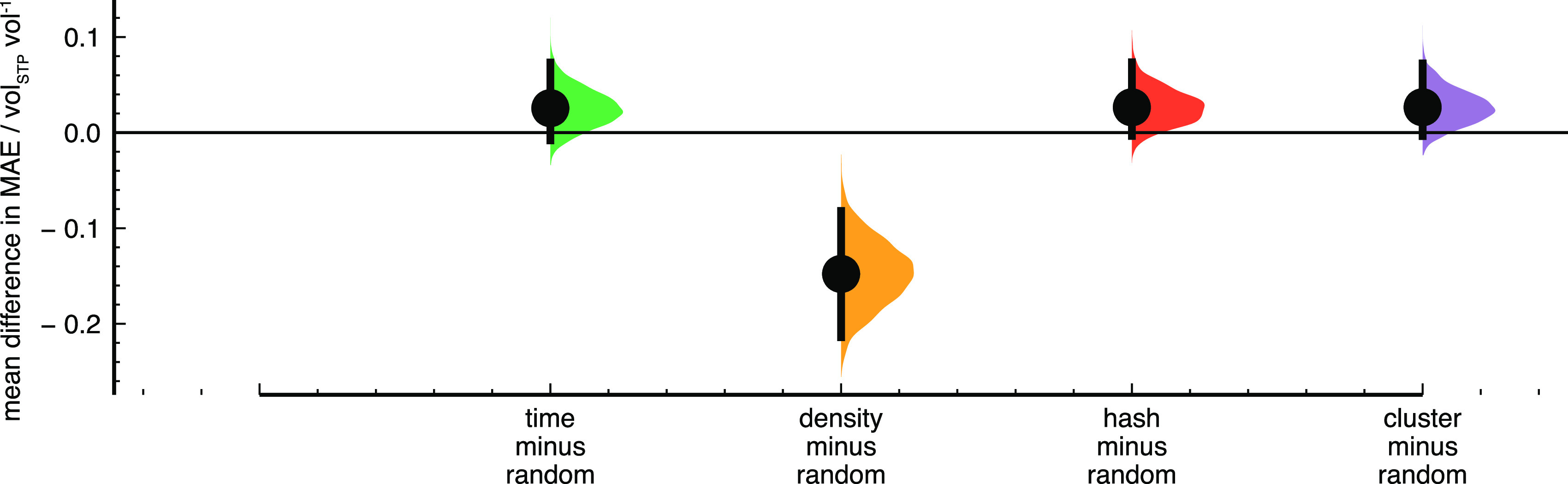
Bootstrapped mean difference in mean absolute error for out-of-domain predictions as a function of the splitting strategy. We tuned gradient boosted decision tree models using the different feature sets implemented in mofdscribe (currently including histograms of persistence diagrams, AMD, geometric properties, APRDF) using different featurizers on the CoRE MOF data set to predict the methane deliverable capacity and evaluate the performance on the ARABG data set. We ran every experiment around 20 times and then computed bootstrapped effect sizes with respect to the random split performance.

**Figure 11:**
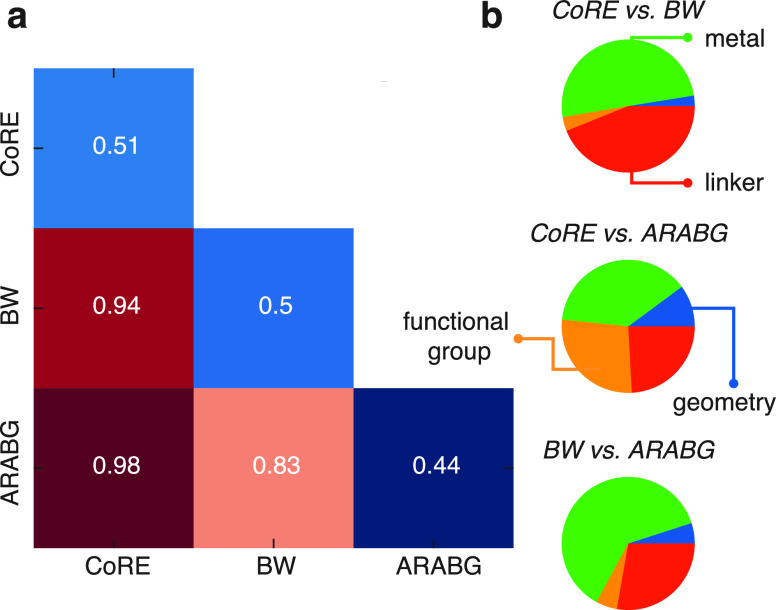
Adversarial validation across different MOF data sets. For this experiment, we reuse the data sets as provided by Moosavi et al.^21^ (i.e., using the same features and labels) and use adversarial validation to estimate how difficult it is for a model to distinguish the data sets. **a** The heatmap shows the area under the receiver-operating characteristic curve (ROC-AUC). **b** Highlights the feature scopes (computed using the Gini impurity decrease estimate) that are most important for the model distinguishing the data sets.

**Figure 12:**
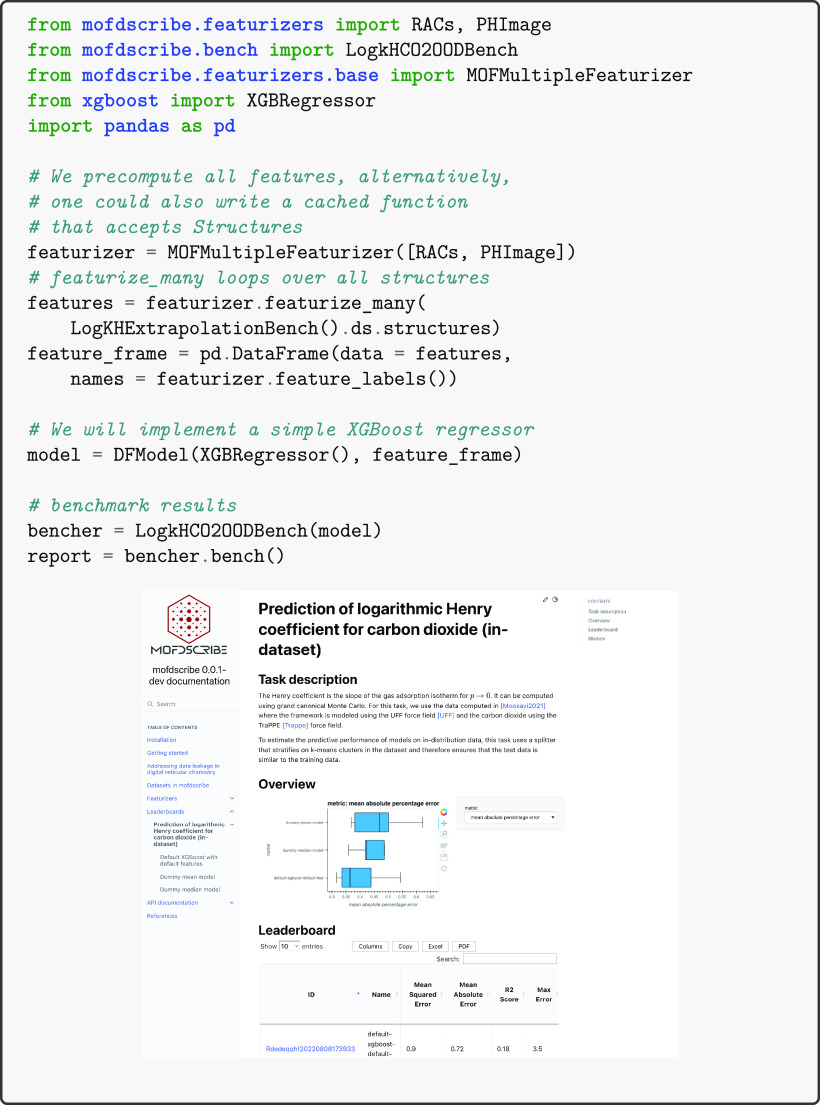
Code for submission to the leaderboard and exemplary screenshot of a leaderboard page.

## Data Availability

Data used in this work are available via the mofdscribe package. The new data set of predicted properties derived from grand canonical Monte Carlo simulations reported with this work is available on the MaterialsCloud^118^ (10.24435/materialscloud:qt-cj) and has been integrated with existing data from the QMOF Database via the Materials Project’s MPContribs interface^119,120^ (10.17188/mpcontribs/1883597). Code availability: The most recent information about the tools is assembled under https://mof.world. The mofdscribe library is available on GitHub (https://github.com/kjappelbaum/mofdscribe). Documentation for the package is available on ReadTheDocs (https://mofdscribe.readthedocs.io/). The graph hashes are implemented in the structuregraph-helpers package, which is also available on GitHub (https://github.com/kjappelbaum/structuregraph-helpers). For encoding (and decoding) of elemental properties, we developed a dedicated library, element-coder, which is also available on GitHub (https://github.com/kjappelbaum/element-coder). The moffragmentor package is also available on GitHub (https://github.com/kjappelbaum/moffragmentor). The AiiDA simulation workflows are based on the ones implemented in the aiida-lsmo package (https://github.com/lsmo-epfl/aiida-lsmo). The topological data analysis workflows are based on moleculetda, available on GitHub (https://github.com/a1k12/moleculetda).

## Appendix: Case Studies

**Case Study 1: The impact of data leakage**

One can argue that in any large data set there will be a few errors. However, the presence of duplicates can cause serious issues for model evaluation.^19^ In this case study, we show that for reticular chemistry, this is a very serious problem.

Using increasingly general (i.e., more structures are considered as duplicates) definitions of duplicates (Figure 7), we analyze how many matching structures we find in the all-solvents removed (ASR) subset of the CSD MOF subset (2019), and the CoRE and QMOF databases. The inverse cumulative histograms below plot how often we find *n* or more identical hashes (e.g., at *n* = 10, the count represents the number of structures with 10 or more identical hashes). The “graph” strategy considers all structures which share the same connectivity and atom types as identical. The “undecorated graph” (undec. graph) strategy does not consider the atom types. The “scaffold” strategy removes all functional groups, solvents, hydrogens, and terminal atoms from consideration (formally, all subgraphs connected via bridges). Again, we can also remove the atom types from consideration. As expected, we see an increase in the larger number of duplicate counts from left to right.

This analysis shows that, for instance, the Co-CPO-74 structure appears 114 times in the ASR CSD MOF subset. This implies that this structure will likely appear both in the training and test set. Importantly, this is not the only structure with many duplicates; there are on the order of 100 structures in which the structure graph appears more than ten times in the CoRE MOF database—and hence do not contribute to a meaningful measure of the generalization performance of the model.

Analyzing a database of in-silico assembled MOF structures (BW)^36^ we find notably many scaffold duplicates: Among nearly 20000 structures we only find 1584 unique undecorated scaffolds. Many in-silico MOF assembly approaches enumerate all possible combinations of building blocks, nets, and functional groups. While this approach can give a lot of detailed insights into regions of chemical space, it will also give rise to many very similar structures that can lead to the violation of the assumption of independence between training and test set. To illustrate this, we can simulate a thousand random train/test splits (as commonly done) and measure how often a scaffold occurs both in the training and test set. For practical train/test ratios, the majority of scaffolds (e.g., 55% for a train/test ratio of 0.8/0.2) will be found in both training and test set.

To showcase the potential impact of such data leakage on model evaluation, we computed learning curves (for CO_2_ Henry coefficients in the BW database as implemented in mofdscribe) for two deduplication levels: No deduplication, and removal of identical undecorated scaffolds. If there would not have been any data leakage, the learning curves should be similar. However, for the deduplicated data sets we observe that the initial learning is faster (presumably because of a higher information density in deduplicated data sets) but much lower than for the data set containing undecorated scaffold duplicates. The increase in performance we observe for the data set with duplicates is most likely caused by data leakage, and the test set contains many structures that are only marginally different from the training set, and hence if we remove these duplicates from our training and test, our error *worsens* significantly. Depending on the strictness of the duplicate definition (Figure 7) one might see—with the same train and test set sizes—drastically larger errors. Moreover, it is important to realize that in our case study, the removal of duplicates increased the learning (steeper learning curves; in fact, this can sometimes lead to better models) and led to a more faithful measure of generalization performance.

**Case Study 2: The impact of splitting strategies**

To investigate the impact of splitting strategies, we train models on experimental data (for which we can also perform a time-based split) and then evaluate how well the models generalize to hypothetical materials.

We trained the gradient boosted decision tree models using the default feature set of CoRE data set in mofdscribe (currently including histograms of persistence diagrams, AMD, geometric properties, APRDF) to predict the methane deliverable capacity. For all experiments, we remove duplicates, i.e., materials with identical structure graphs. We then optimize hyperparameters of gradient boosted decision trees using Bayesian optimization on the validation set and train the model using different splitting strategies, always keeping the train/validation/test ratios fixed. The figure uses the random split as the control group and computes bootstrapped mean effect sizes for the different splitting approaches. We see that all splitting strategies lead to models with different generalization performances (better in all cases except for the density-based split) than the random control group.

This impact of the splitting strategy also indicates the need to quantify the difficulty of a given validation split. As one method to do so, mofdscribe implements adversarial validation, which quantifies how easily a machine learning model can distinguish the train from the test set.

In Figure 11, panel **a** shows the adversarial validation scores for the data sets considered in Moosavi et al.^21^ For the entries on the diagonal, we considered a random split into two equally sized parts. Scores closer to one indicate that the data sets are easily distinguishable. In this case, we see that a model can easily distinguish the data sets—in particular, the experimental ones from the in-silico assembled ones. Therefore, we cannot expect a model to necessarily generalize in this setting. In panel **b** we see that the analysis of the feature importance can reveal which features the model used to distinguish the data sets. Removing those features can help to mitigate data-drift features or also help to guide the generation of new materials that can mitigate those biases (or remove materials that are dissimilar from the target distribution, i.e., have a ROC-AUC score greater than 0.5). When we group the features into scopes, as in Moosavi et al.,^21^ we find that the dominating differences across databases vary. While linker feature contributions do not play a major role in distinguishing structures from the BW and CoRE databases, they do play an important role in distinguishing structures from the CoRE and ARABG database. BW denotes a database of hypothetical MOFs assembled by Boyd and Woo,^35^ and ARABG abbreviates a database of hypothetical MOFs assembled by Anderson et al.^37^

**Case Study 3: Creating a new model and submitting it to the leaderboard**

We implement a full modeling pipeline from featurization to benchmarking in the following code (Figure 12). Note, however, that in practice, there will be additional steps that tune features and model hyperparameters.

More examples, including one on an experimental data set, can be found in in the GitHub repository (https://github.com/kjappelbaum/mofdscribe/tree/main/examples). The examples can be run on Google Colab (e.g., https://colab.research.google.com/github/kjappelbaum/mofdscribe/blob/main/examples/build_model_using_mofdscribe.ipynb).

## Supporting Information Available

Computational details of the reference data set and implementation details for deduplication, featurization, data splitters, MOF fragmentation, and leaderboard (PDF)
Transparent Peer Review report available (PDF)

## Overview of Implemented Featurizers. An up-to-date list can always be found in the online documentation

AccessibleVolume: accessible volume, computed using the zeo++ code.^66,67^
AMD: generalization of the average-minimum distance approach proposed by Widdowson et al.^57^
APRDF: generalization of the atomic-property labeled radial distribution function proposed in Fernandez et al.^121^
Asphericity: shortcut for the asphericity descriptor^122,123^ implemented in RDKit.^62^
AtomCenteredPH: atom-centered persistent homology. Analogous to the approach reported by Jiang et al.^124^
DiskLikeness: molecular descriptors computed based on principle moment of inertia, computed using RDKit.^62^ Descriptor proposed by Wirth et al.^125^ as a measure of ligand shape.
Eccentricity: shortcut for the eccentricity descriptor^122,126^ implemented in RDKit.^62^
EnergyGridHistogram: energy gird histograms, computed using RASPA,^113^ as proposed by Bucior et al.^127^
GuestCenteredAPRDF: This featurizer builds on the APRDF featurizer, but instead of using the correlations between all atoms, it only considers the ones between the guest and all host atoms (within some cutoff distance).
Henry: Henry coefficient, as computed using RASPA.^113^
InertialShapeFactor: shortcut for the inertial shape factor descriptor^122,126^ implemented in RDKit.^62^
LSOP: local structure order parameters (LSOP), modified approach from Zimmermann and Jain^56^ Here we place a site at the center of mass and then compute the LSOPs around this site. In this way, we attempt to capture the shapes of full building blocks.
NConf20: molecular flexibility descriptor based on the number of energetically accessible conformers. Based on implementation of Wicker and Cooper^128^ using RDKit.^62,129^
NPR1: shortcut for the normalized principal moments ratio 1 (= I_1_/I_3_) descriptor^130^ in RDKit.^62^
NPR2: shortcut for the normalized principal moments ratio 2 (= I_2_/I_3_) descriptor^130^ in RDKit.^62^
PairwiseDistanceHist: histogram of pairwise distances between atoms in a molecule/structure
PairwiseDistanceStats: statistics of pairwise distances between atoms in a molecule/structure
PartialChargeHistogram: histogram of partial charges computed with a charge equilibration strategy (EqEq).^131^
PartialChargeStats: statistics of partial charges computed with a charge equilibration strategy (EqEq).^131^
PHHist: (2D) histogram of persistence diagrams, computed based on developments by Krishnapriyan et al.,^72,73^ using the dionysus and diode codes (latter being a Python binding to parts of CGAL(132)).
PHImage: vectorization of persistence diagrams as persistence image^133^ computed based on developments by Krishnapriyan et al.,^72,73^ using the dionysus and diode codes (latter being a Python binding to parts of CGAL(132)).
PHStats: statistics of persistence diagrams, computed based on developments by Krishnapriyan et al.,^72,73^ using the dionysus and diode codes (latter being a Python binding to parts of CGAL(132)).
PHVect: vectorization of persistence diagrams using Gaussian mixture models,^74,75^ computes using the pervect^134^ library.
PMI1: first principle moment of inertia, computed with RDKit.^62^
PMI2: second principle moment of inertia, computed with RDKit.^62^
PMI3: third principle moment of inertia, computed with RDKit.^62^
PoreDiameters: pore radii, computed with zeo++^67^
PoreSizeDistribution: histogram of pore sizes, computed with zeo++.^67^ Has been used in Pinheiro et al.^68^
PriceLowerBound: lower bound for the MOF price based on elemental prices (surrogate for chemistry and useful as a screening filter).
RACS: revised autocorrelation functions, as proposed by Janet and Kulik^54^ and applied to MOFs by Moosavi et al.^21^
RadiusOfGyration: shortcut for the radius of gyration descriptor^126^ implemented in RDKit.^62^
RayTracingHistogram: histograms of ray lengths passed through the unit cell, computed using zeo++(67) Proposed by Jones et al.^69^
RodLikeness: molecular descriptors computed based on principle moment of inertia, computed using RDKit.^62^ Descriptor proposed by Wirth et al.^125^ as a measure of ligand shape.
BUMatch: minimum root-mean-squared-distance between the connecting site structure of the building blocks and the “ideal” one in different nets.
SphereLikeness: molecular descriptors computed based on principle moment of inertia, computed using RDKit.^62^ Descriptor proposed by Wirth et al.^125^ as a measure of ligand shape.
SpherocityIndex: shortcut for the spherocity descriptor^122^ implemented in RDKit.^62^
SurfaceArea: (probe accessible) surface areas, as computed using zeo++(67)
VoxelGrid: 3D voxel representations of the structure. Similar to Hung et al.^80^ and Cho and Lin^81^
