# Supplementary material for: An Ecosystem for Digital Reticular Chemistry
Source: ACS Cent Sci. 2023 Mar 10;9(4):563–81. doi: 10.1021/acscentsci.2c01177 (PMC10141625; doi:10.1021/acscentsci.2c01177)
Supplement: Supplementary file 1 — oc2c01177_si_001.pdf [file oc2c01177_si_001.pdf]

Supplementary Information for: An ecosystem for digital reticular chemistry

Kevin Maik Jablonka[1], Andrew S. Rosen[2,3,4], Aditi S. Krishnapriyan,[5,6,7] Berend Smit[1,✉]

[1]Laboratory of Molecular Simulation (LSMO), École Polytechnique Fédérale de Lausanne (EPFL), Sion, Switzerland. [2]Department of Materials Science and Engineering, University of California, Berkeley, CA, 94720, USA
[3]Miller Institute for Basic Research in Science, University of California, Berkeley, Berkeley, CA, 94720, USA
[4]Materials Science Division, Lawrence Berkeley National Laboratory, Berkeley, CA, 94720, USA
[5]Department of Chemical and Biomolecular Engineering, University of California, Berkeley, CA, 94720, USA
[6]Department of Electrical Engineering and Computer Science, University of California, Berkeley, CA, 94720, USA
[6]Computational Research Division, Lawrence Berkeley National Laboratory, Berkeley, CA, 94720, USA

✉ berend.smit@epfl.ch

Contents

1 Growth of machine learning for reticular chemistry and porous materials S2

2 Gas adsorption reference dataset S3
2.1 Computed properties S3
2.2 Dataset description S6

3 Duplicates S18

4 Featurizers S19
4.1 Addition of aggregations S19

5 Graph hashes S20

6 Splitters S21
6.1 Grouped and stratified holdout splits S21
6.2 Case studies S21

7 MOF fragmentation S24

8 Leaderboard S26

Supplementary Note 1 Growth of machine learning for reticular chemistry and porous materials

To illustrate the importance of machine learning for reticular chemistry and porous materials we conducted a literature survey using Scopus, interfaced via `pybibliometrics`.[1] We used `TITLE-ABS-KEY(("reticular chemistry" OR "metal-organic framework" OR "covalent-organic framework" or "zeolite") AND ("machine learning" OR "neural network" OR "data-driven" OR "deep learning" OR "random forest" OR "gradient boost" OR "support vector" OR "regression" OR "recommendation system" OR "inverse design" OR "recommender system" OR "active learning" OR "bayesian optimization"))` as query and plot in Figure 1 the number of publications aggregated per year (excluding 2023, which already counted 7 matches at the time we last performed this survey, December 12, 2022).

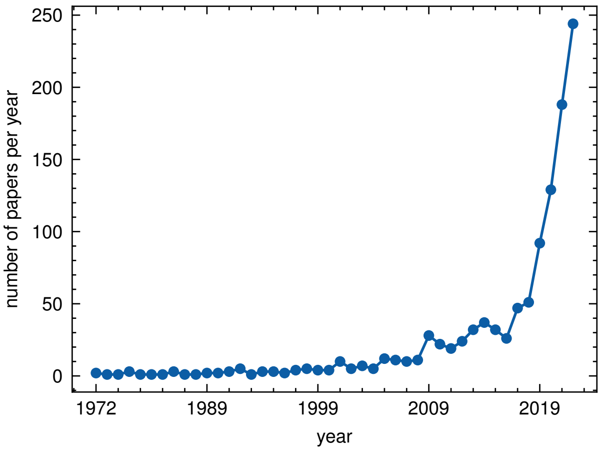

Supplementary Figure 1 | Count of publications aggregated per year that mention some concept of reticular chemistry or porous materials besides machine learning concepts.

Supplementary Note 2 Gas adsorption reference dataset

Note that this dataset contains a significant fraction of zeros for cases in which the simulation was skipped because they were deemed inaccessible to the guest following geometric analysis. This is because the QMOF dataset focused, for reasons of computational cost, on the subset of MOFs with small unit cells. Additionally, it is important to realize that the MOF subset of the CSD[2] contains a large fraction of nonporous materials (best described as coordination polymers).

2.1 Computed properties

We used the DDEC6 charges[3–6] from the PBE-D3(BJ) calculations as provided with the QMOF database. We always described the framework using the UFF[7] forcefield, applying analytical tail-corrections[8] for the contributions after the cutoff of 12 Å. For zeo++[9], we always used 100,000 samples for the calculation of the probe-occupiable volume and 100 $Å^{-3}$ samples for the computation of the blocked pockets.
CO_2 and N_2 isotherms were sampled using the algorithm described in Ongari et al.[10] For other isotherms, we used a fixed grid of pressure points. We always employed 100,000 cycles with the RASPA code for the computation of the Henry coefficients.[11] The additional simulation-specific settings are detailed in the following Supplementary Tables. The full provenance graph can be downloaded on the MaterialsCloud (10.24435/materialscloud:qt-cj).

Supplementary Table 1 | Simulation parameters for CO_2 isotherms (and Widom insertions).

parameter	*value*
force field guest	TraPPE[12]
saturation density / mol L^{-1}	21.2
probe radius / Å	1.525
T / K	300
initialization cycles	1000
production cycles	10000
p sampling precision	0.1
max distance between p points / bar	5
lowest pressure point / bar	0.001
largest pressure point / bar	30

Supplementary Table 2 | Simulation parameters for N_2 isotherms (and Widom insertions).

parameter	*value*
force field guest	TraPPE[12]
saturation density / mol L^{-1}	28.3
probe radius / Å	1.655
T / K	300
initialization cycles	1000
production cycles	10000
p sampling precision	0.1
max distance between p points / bar	5
lowest pressure point / bar	0.001
largest pressure point / bar	30

Supplementary Table 3 | Simulation parameters for H_2 isotherms (and Widom insertions).

parameter	*value*
force field guest	MDT+DL (as described in Bucior et al.[13], i.e., dispersion from Michels et al.[14] and charges from Darkrim and Levesque[15])
saturation density / mol L^{-1}	35.4
probe radius / Å	1.48
initialization cycles	3000
production cycles	3000
temperature grid / K	77, 198, 298
pressure grid / bar	1.0, 5.0, 25, 50, 75, 100

Supplementary Table 4 | Simulation parameters for CH_4 isotherms (and Widom insertions).

parameter	*value*
force field guest	TraPPE[16]
saturation density / mol L^{-1}	26.34
probe radius / Å	1.865
T / K	298
initialization cycles	1000
production cycles	10000
pressure grid / bar	1.0, 5.8, 20, 35, 50, 65

Supplementary Table 5 | Simulation parameters for O_2 isotherms (and Widom insertions). Based on settings chosen in Moghadam et al. [17]

parameter	*value*
force field guest	TraPPE[18]
saturation density / mol L^{-1}	71.3
probe radius / Å	1.51
T / K	298
initialization cycles	5000
production cycles	5000
pressure grid / bar	1, 5, 10, 20, 30, 50, 80, 100, 140, 200

Supplementary Table 6 | Simulation parameters for Xe Widom insertions.

parameter	*value*
force field guest	BOATO[19]
saturation density / mol L^{-1}	22.4
probe radius / Å	1.985

Supplementary Table 7 | Simulation parameters for Kr Widom insertions.

parameter	*value*
force field guest	BOATO[19]
saturation density / mol L^{-1}	29.0
probe radius / Å	1.83

Supplementary Table 8 | Simulation parameters for water Widom insertions.

parameter	*value*
force field guest	TIP4P/2005[20]
saturation density / mol L^{-1}	53.3
probe radius / Å	1.58

Supplementary Table 9 | Simulation parameters for H_2S Widom insertions.

parameter	*value*
force field guest	ESP-MM[21]
saturation density / mol L^{-1}	26.9
probe radius / Å	1.74

For the process simulations, we used very simplified models of a temperature–pressure swing process[22] in which we assume a constant heat capacity for all materials, which is known not to be a correct approximation.[23]

2.2 Dataset description

The following Supplementary Figures show the distributions of the computed properties.

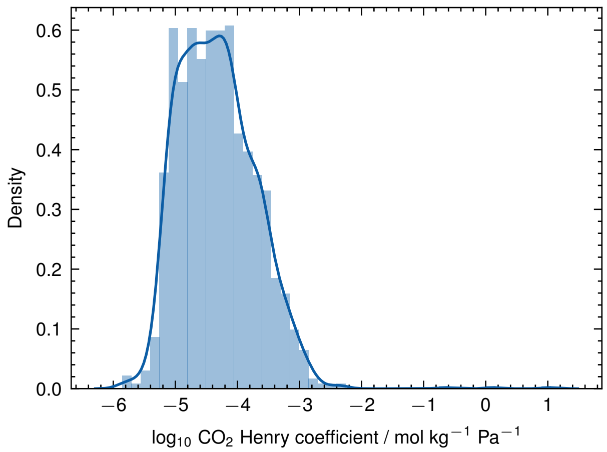

Supplementary Figure 2 | Distribution of $\log_{10}$ CO_2 Henry coefficient / mol kg^{-1} Pa^{-1}.

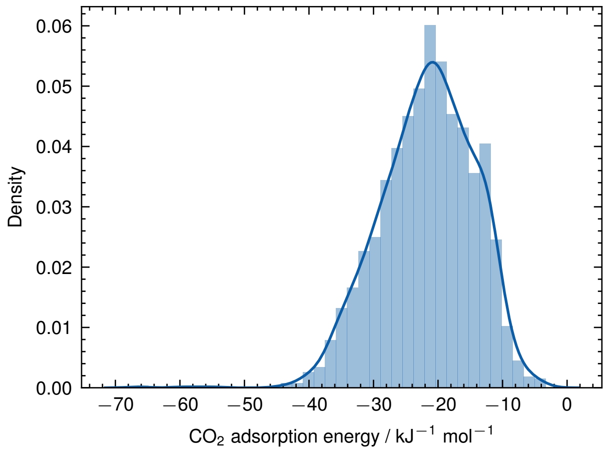

Supplementary Figure 3 | Distribution of $\log_{10}$ CO_2 adsorption energy / kJ mol^{-1}.

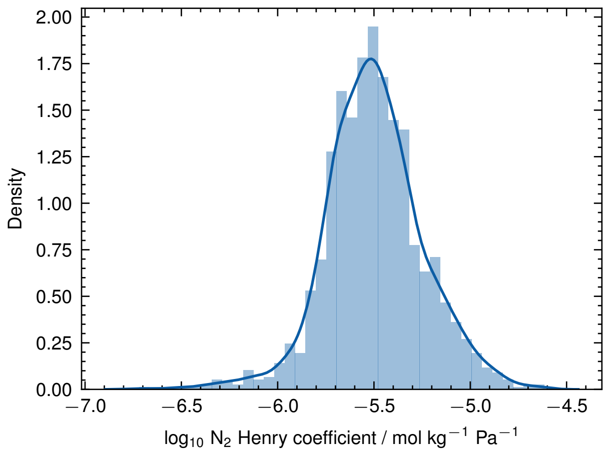

Supplementary Figure 4 | Distribution of $\log_{10}$ N_2 Henry coefficient / mol kg^{-1} Pa^{-1}.

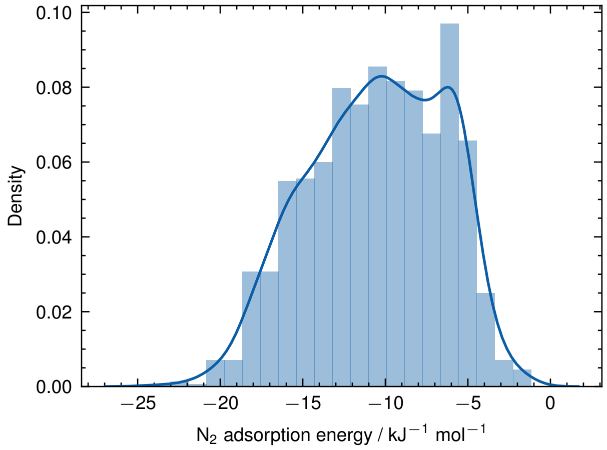

Supplementary Figure 5 | Distribution of $\log_{10}$ N_2 adsorption energy / kJ mol^{-1}.

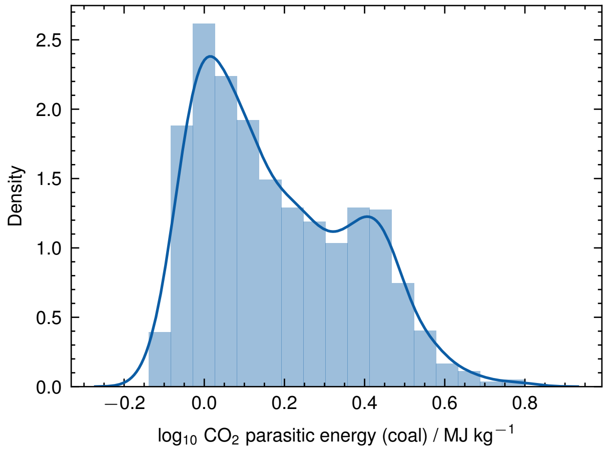

Supplementary Figure 6 | Distribution of $\log_{10}$ CO_2 parasitic energy (coal) / MJ kgl^{-1}.

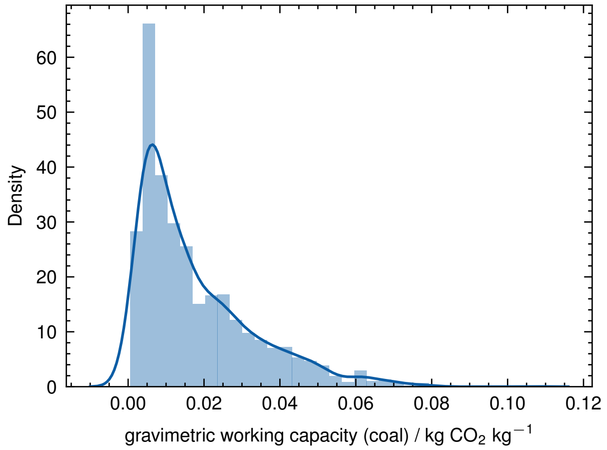

Supplementary Figure 7 | Distribution of $\log_{10}$ CO_2 gravimetric working capacity (coal) / kg CO_2 kg^{-1}.

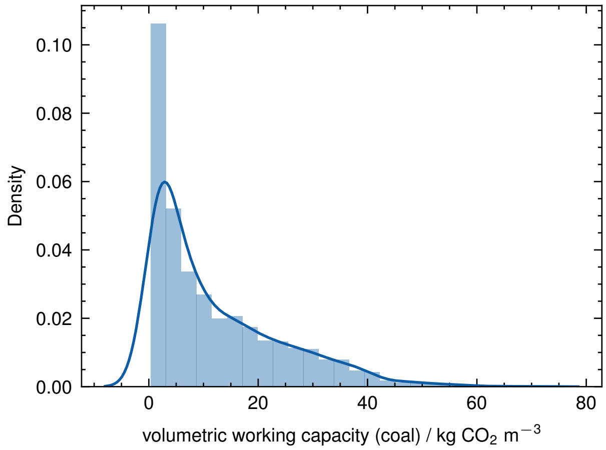

Supplementary Figure 8 | Distribution of $\log_{10}$ volumetric working capacity (coal) / kg CO_2 m^{-3}.

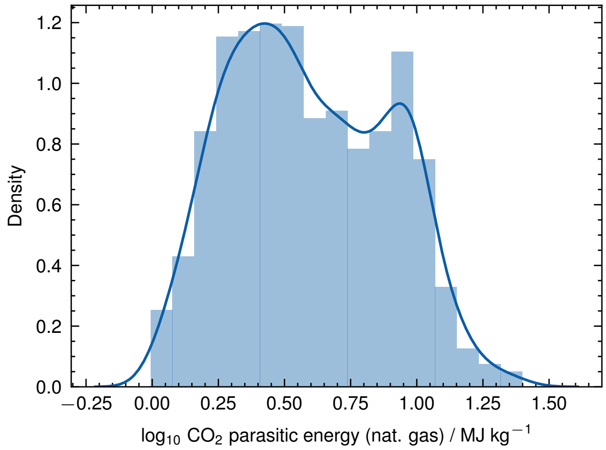

Supplementary Figure 9 | Distribution of $\log_{10}$ CO_2 parasitic energy (nat. gas) / MJ kgl^{-1}.

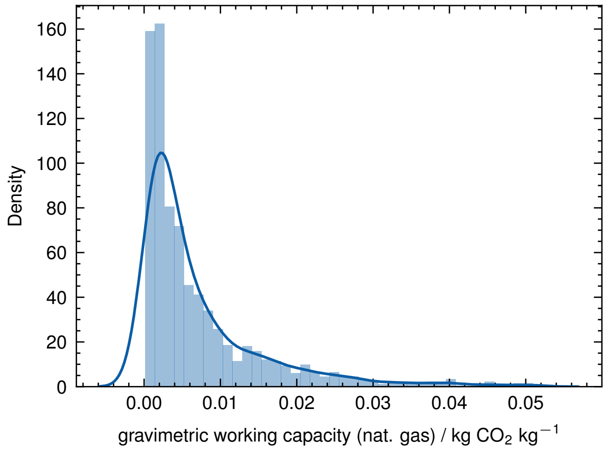

Supplementary Figure 10 | Distribution of $\log_{10}$ CO_2 gravimetric working capacity (nat. gas) / kg CO2 kg^{-1}.

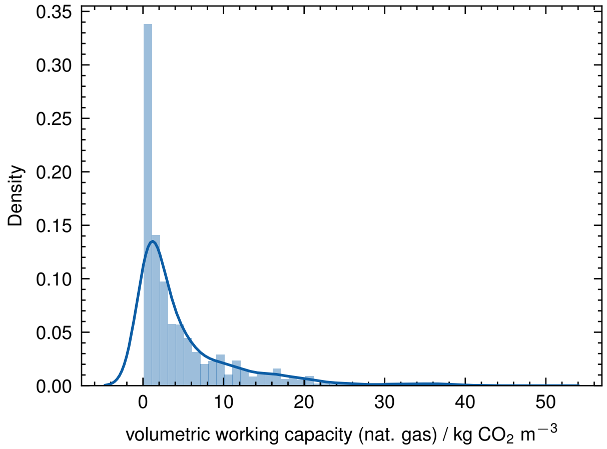

Supplementary Figure 11 | Distribution of $\log_{10}$ volumetric working capacity (nat. gas) / kg CO_2 m^3.

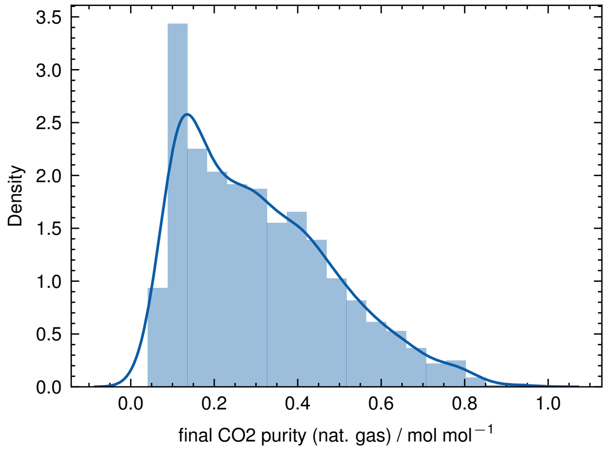

Supplementary Figure 12 | Distribution of $\log_{10}$ final CO_2 purity (nat. gas) / mol mol^{-1}.

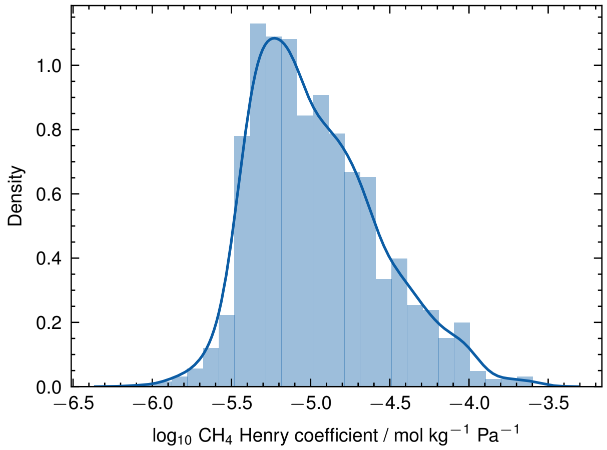

Supplementary Figure 13 | Distribution of $\log_{10}$ CH_4 Henry coefficient / mol kg^{-1} Pa^{-1}.

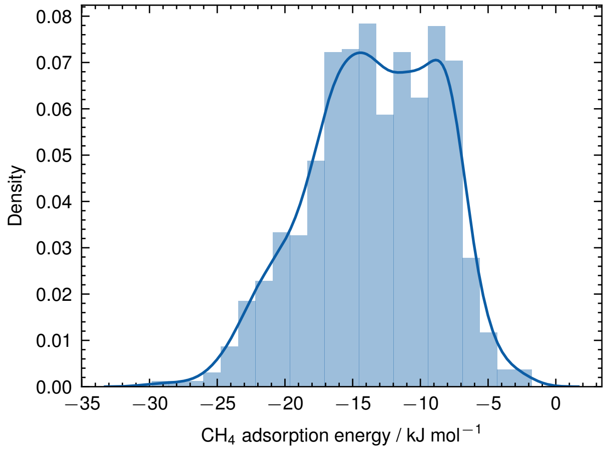

Supplementary Figure 14 | Distribution of $\log_{10}$ CH_4 adsorption energy / kJ mol^{-1}.

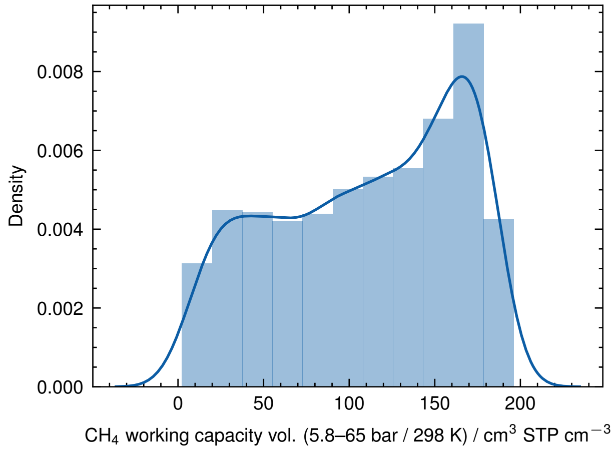

Supplementary Figure 15 | Distribution of $\log_{10}$ CH_4 working capacity vol. (5.8-65 bar/298 K) / cm$^3_{STP}$ cm^{-3}.

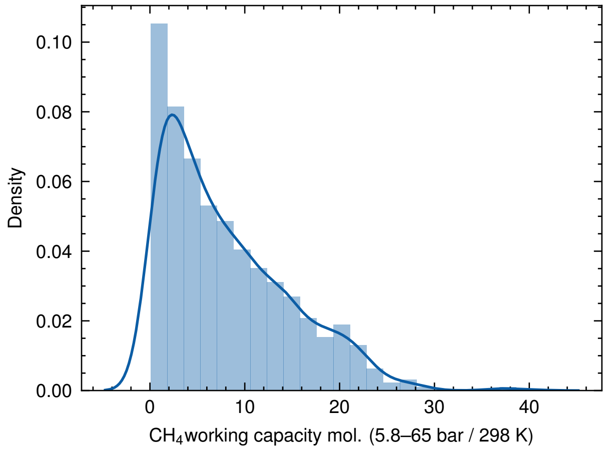

Supplementary Figure 16 | Distribution of $\log_{10}$ CH_4 working capacity (5.8-65bar/298K) / mol kg^{-1}.

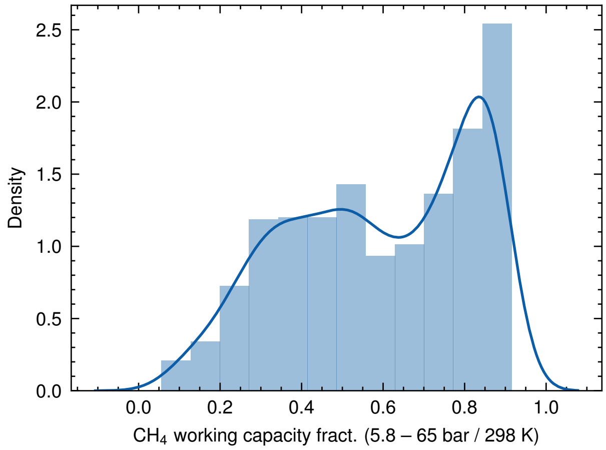

Supplementary Figure 17 | Distribution of $\log_{10}$ CH_4 working capacity fract. (5.8-65 bar/298 K).

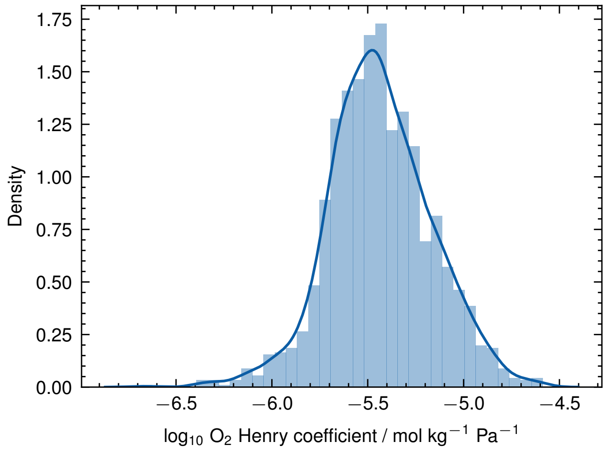

Supplementary Figure 18 | Distribution of $\log_{10}$ O_2 Henry coefficient / mol kg^{-1} Pa^{-1}.

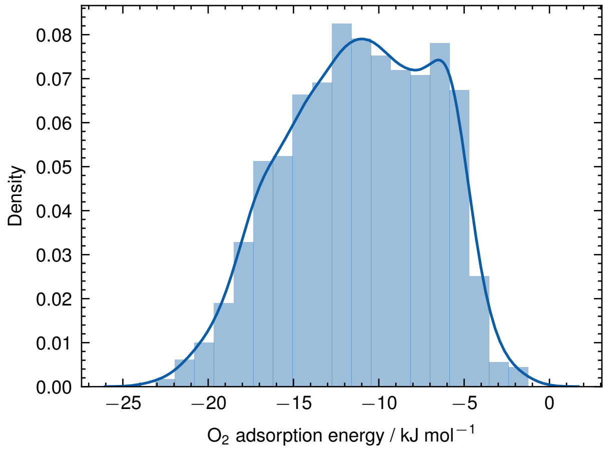

Supplementary Figure 19 | Distribution of $\log_{10}$ O_2 adsorption energy / kJ mol^{-1}.

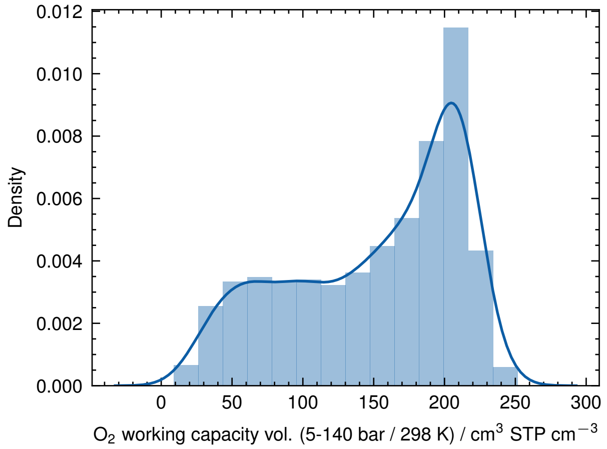

Supplementary Figure 20 | Distribution of $\log_{10}$ O_2 working capacity (5-140 bar/298 K) / cm^3_{STP} cm^{-3}.

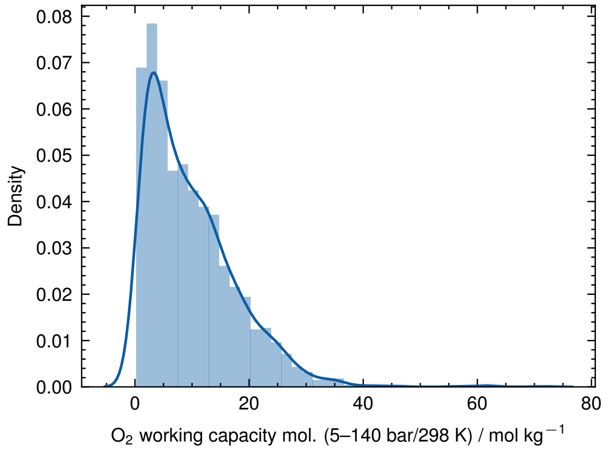

Supplementary Figure 21 | Distribution of $\log_{10}$ O_2 working capacity (5-140 bar/298 K) / mol kg^{-1}.

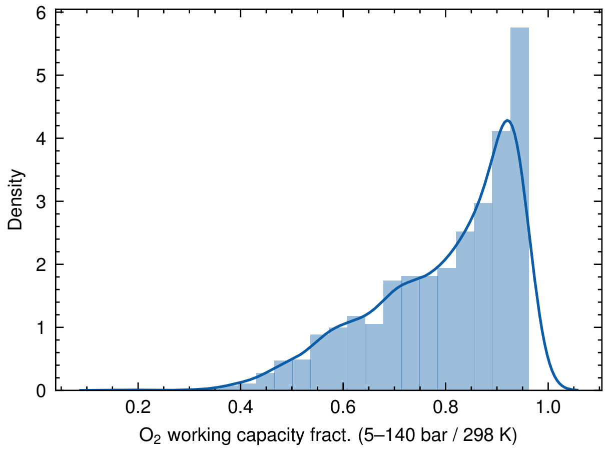

Supplementary Figure 22 | Distribution of $\log_{10}$ O_2 working capacity (5-140 bar/298 K).

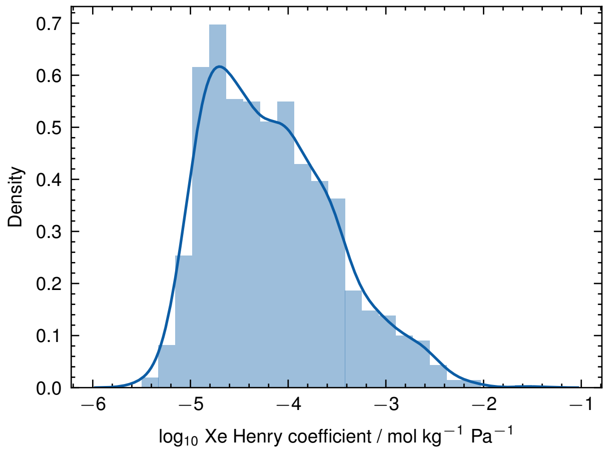

Supplementary Figure 23 | Distribution of $\log_{10}$ Xe Henry coefficient / mol kg^{-1} Pa^{-1}.

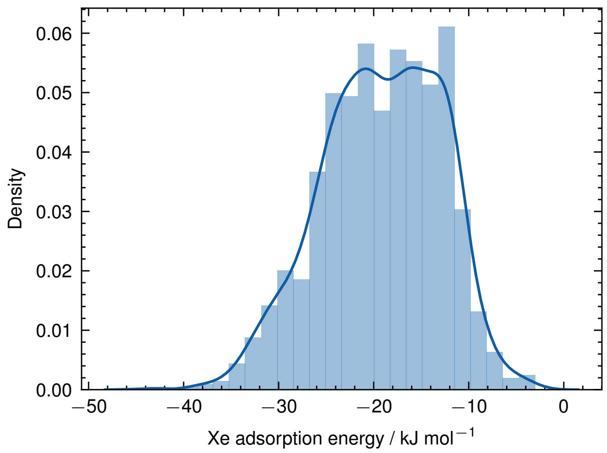

Supplementary Figure 24 | Distribution of $\log_{10}$ Xe adsorption energy / kJ mol^{-1}.

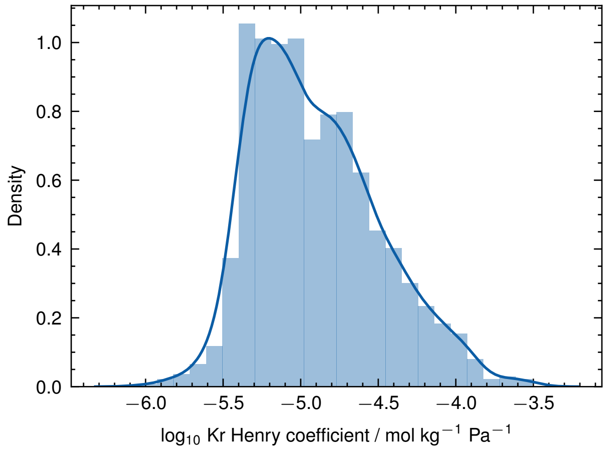

Supplementary Figure 25 | Distribution of $\log_{10}$ Kr Henry coefficient / mol kg^{-1} Pa^{-1}.

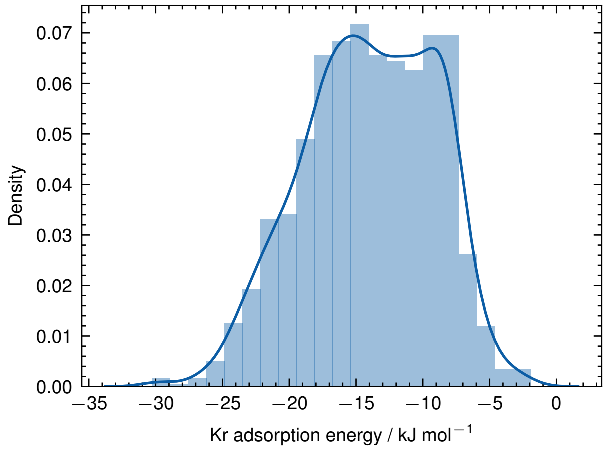

Supplementary Figure 26 | Distribution of $\log_{10}$ Kr adsorption energy / kJ mol^{-1}.

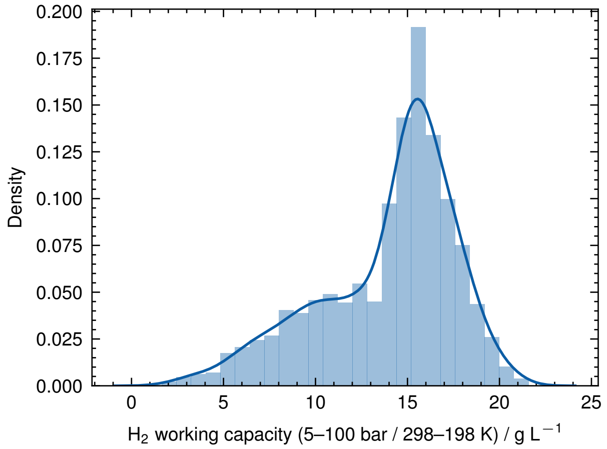

Supplementary Figure 27 | Distribution of $\log_{10}$ H_2 working capacity (5-100 bar/298-198 K) / g L^{-1}.

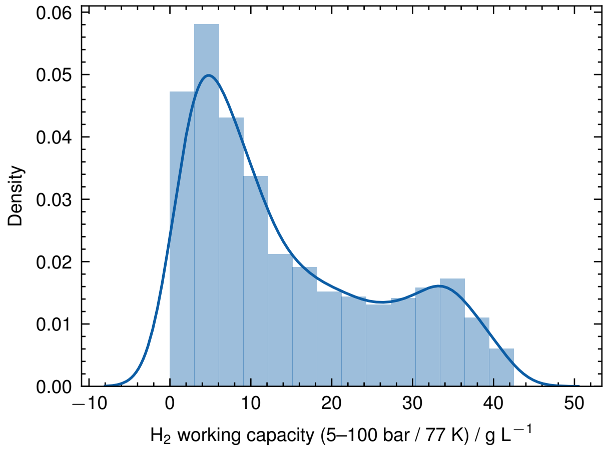

Supplementary Figure 28 | Distribution of $\log_{10}$ H_2 working capacity (5-100 bar/77 K) / g L^{-1}.

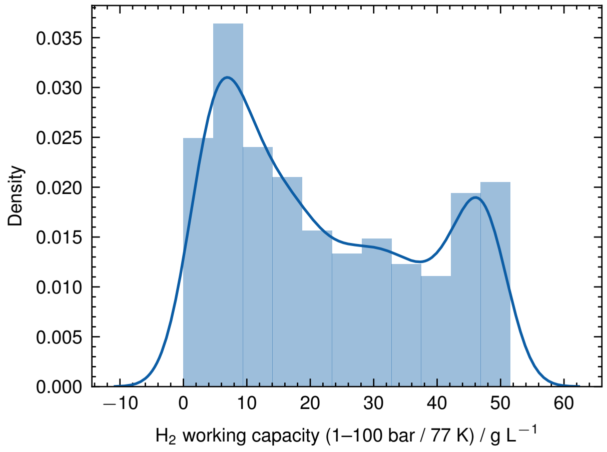

Supplementary Figure 29 | Distribution of $\log_{10}$ H_2 working capacity (1-100 bar/77 K) / g L^{-1}.

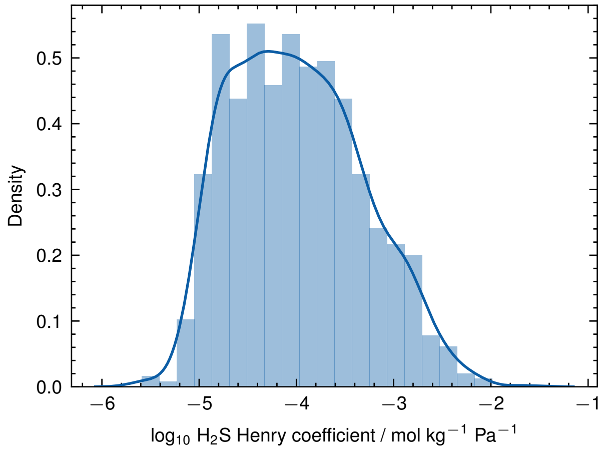

Supplementary Figure 30 | Distribution of $\log_{10}$ H_2S Henry coefficient / mol kg^{-1} Pa^{-1}.

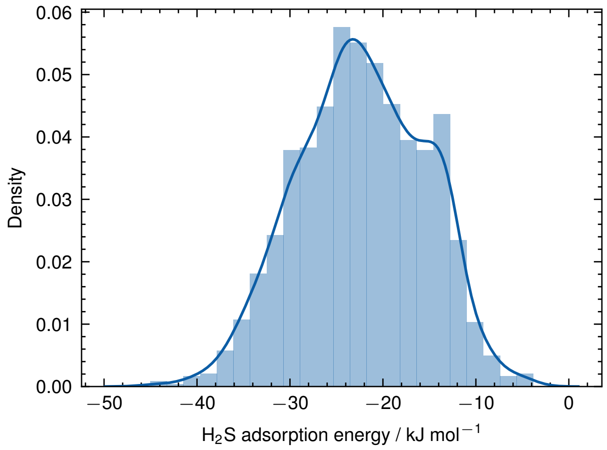

Supplementary Figure 31 | Distribution of $\log_{10}$ H_2S adsorption energy / kJ mol^{-1}.

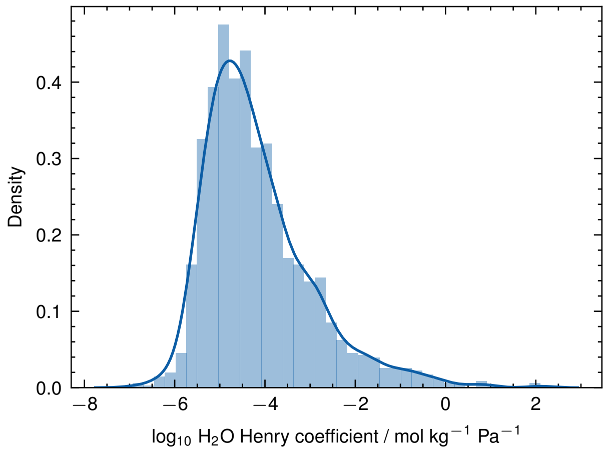

Supplementary Figure 32 | Distribution of $\log_{10}$ H_2O Henry coefficient / mol kg^{-1} Pa^{-1}.

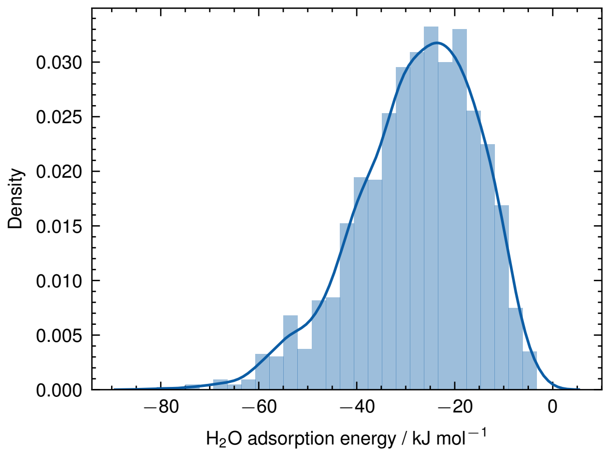

Supplementary Figure 33 | Distribution of $\log_{10}$ H_2O adsorption energy / kJ mol^{-1}.

Supplementary Note 3 Duplicates

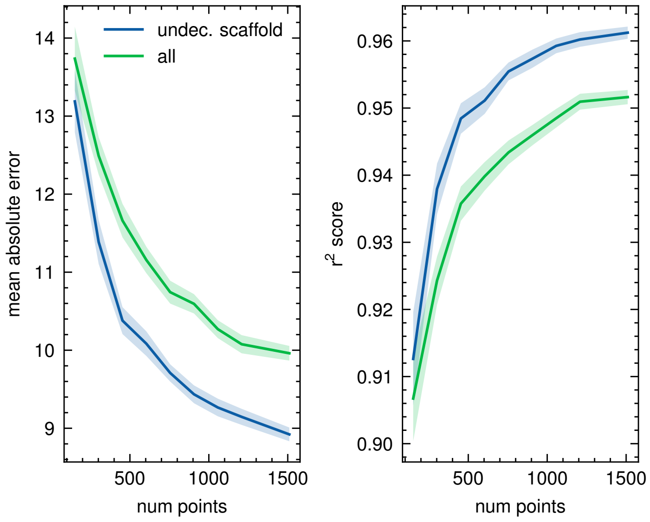

Supplementary Figure 34 | Learning curves with all data points and only unique undecorated scaffolds (BW dataset and CH_4 deliverable capacity as the target, using XGBoost regressor on the default dataset). Shaded area indicates 95 % standard intervals. For this case study, dropping of undecorated scaffold duplicates leads to improved performance metrics.

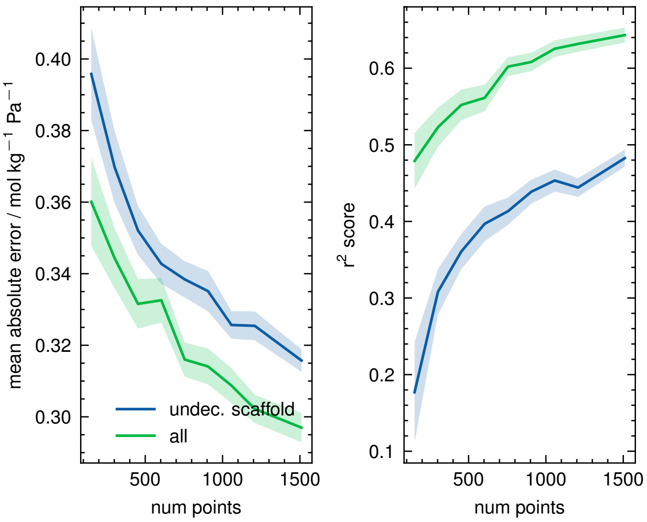

Supplementary Figure 35 | Learning curves with all data points and only unique undecorated scaffolds (CoRE dataset and CO_2 Henry coefficient as the target, using XGBoost regressor on the default dataset). Shaded area indicates 95 % standard intervals.

Supplementary Note 4 Featurizers

4.1 Addition of aggregations

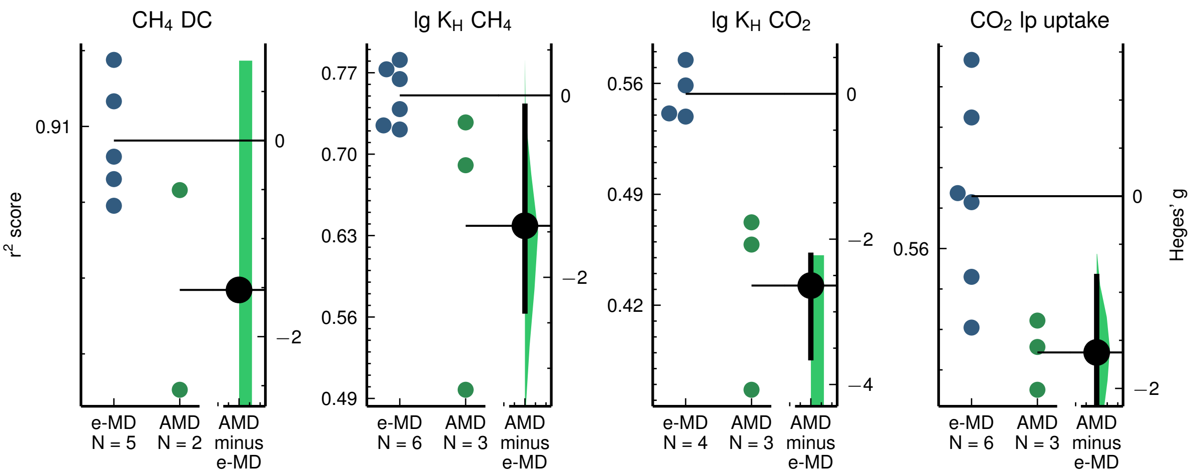

Supplementary Figure 36 | Minimum distance fingerprint with and without additional aggregations. The original average minimum distance fingerprints as proposed by Widdowson et al. aggregate point-wise distance distributions (PDD) using (weighted) averages (top row). In mofdscribe, users can also compute other aggregations such as min, max, and the standard deviation (std, bottom row, e-MD). To ensure fair comparison we optimize the *full* pipeline (including pre-processing and the model) using automated machine learning.[24] The plots visualize the measured model performance and estimated effect sizes in terms of Hedges' g.[25] The blue points always indicate the coefficient of determination (r^2 on a holdout test set, measured on the left axes) of the models trained with additional aggregations (e-MD.) The green ones indicate the coefficients of determination of the models trained with only the mean (AMD) as aggregation. To quantify the effect, we bootstrap the Hedges' g (a suitable effect size metric in the case of little data,[26] shown on the right axes) and show it with a kernel density estimate. In all cases, the addition of chemistry shows very large effects.[27]

Supplementary Note 5   Graph hashes

Weisfeiler and Lehman devised a graph-isomorphism test based on iterative refinement of graph colorings to derive a canonical form.[28] Two non-isomorphic graphs might share the same canonical form; however, if the canonical forms are not identical, the graphs are definitely not isomorphic. Therefore, this test might lead us to identify too many isomorphic graphs. However, since our objective is mostly duplicate removal to avoid data leakage, this is preferable to missing duplicates.

In practice, we encode the periodic crystal graph as a labeled quotient graph (LQG) (labels indicating into which periodic image the bonding extends) but do not consider the directions and edge voltages for the hash derivation. That is, we rely on the fact that two LQGs of the same crystallographic net cannot have non-isomorphic unlabeled quotient graphs (UQGs). Hence, computing a hash of the Weisfeiler-Lehman canonical form of the UQG will yield always lead to too many duplicates, not too few.

Supplementary Note 6 Splitters

Our main objectives for the default settings in the splitter classes are:

- To minimize data leakage. To ensure this, we implement grouped splits. Typically, we group on undecorated scaffold hashes.
- To minimize imbalance effects on the imbalance. To ensure this, we implement stratification. Typically, we stratify on the target (and bin if the target is continuous[1]).

6.1 Grouped and stratified holdout splits

While we can rely on `sklearn`'s implementations for the grouped k-fold cross-validation case, there is no off-the-shelf implementation for the grouped and stratified case (for k-fold cross-validation and partitioning). Therefore, we implement the following algorithm in which we perform the following steps:

- Assign each structure to a group, for instance, based on the undecorated scaffold hashes.
- Aggregate the target property within each group, e.g., using the arithmetic mean.
- Use the aggregated properties for a stratified split
- For each group, add all the members

While this algorithm does not guarantee that we will match the requested train/test ratio, it guarantees grouping and some level of stratification, which are more important objectives for a stringent model evaluation.

6.2 Case studies

Hyperparameter grid

- `colsample_bylevel`: on logarithmic grid from 0.01 to 0.1
- `depth`: integers between 1 and 16
- `iterations` integer between 1 and 10000
- `learning_rate` float on log scale between 0.001 and 0.5
- `l2_leaf_reg`: float between 0.01 and 10
- `random_strength`: float between 0.01 and 10

[1] Note that this is already a weak source of data leakage as we need to consider the full dataset for binning. Also, note that there is still debate on the pros and cons of this approach, see, https://github.com/kjappelbaum/mofdscribe/discussions/242.

- `bagging_temperature`: float between 0.01 and 10

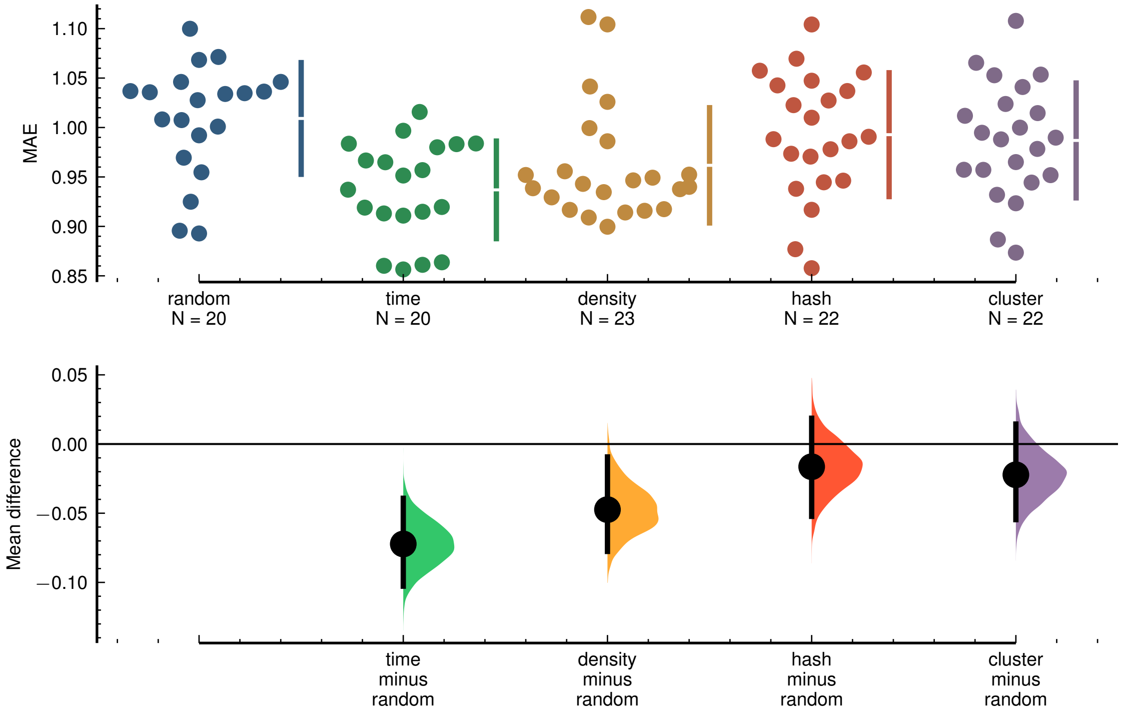

Supplementary Figure 37 | Bootstrapped mean difference in mean absolute error for the prediction of low-pressure CO_2 uptake. The model was trained on the CoRE dataset in `mofdscribe` with the default feature set and tested on the ARABG dataset.

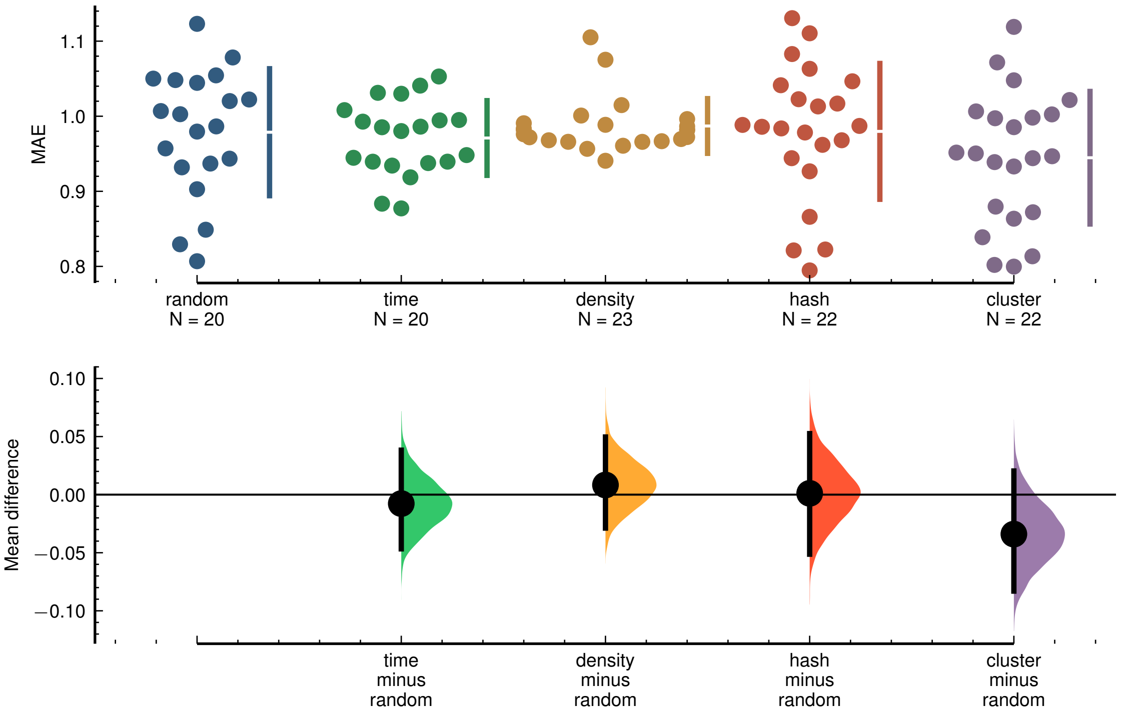

Supplementary Figure 38 | Bootstrapped mean difference in mean absolute error for the prediction of low-pressure CO_2 uptake. The model was trained on the CoRE dataset in `mofdscribe` with the default feature set and tested on the BW dataset.

Supplementary Note 7 MOF fragmentation

The MOF fragmentation algorithm is outlined in Algorithm 1. Therein, we use the following definitions:

- *branching site* is a site that fulfills the following conditions:
  - has at minimum coordination number 3
  - has at least one path with maximum 2 edges that leads to metal and does not contain a bride
  - has at minimum 2 non-metal connections that are not bridges

  If there are multiple neighboring sites selected according to this definition, we pick the one closest to the metal (the fewest number of edges).

- *bridge* is an edge that, when broken, increases the number of connected components
- *connected component* is a connected subgraph that is *not* part of any larger connected subgraph

Data: MOF
Result: fragments and net
unbound_solvent = `locate_unbound_solvent`(*MOF*)
ignored_metals = []
while *potential_metal_in_linker* **do**
  node_candidates = `locate_nodes`(*MOF, unbound_solvent, ignored_metals*)
  bound_solvent = `locate_bound_solvent`(*MOF, node_candidates*)
  linker_candidates = `locate_linkers`(*MOF, node_candidates, bound_solvent*)
  metals_to_ignore, potential_metal_in_linker =
   `check_metal_in_linker`(*MOF, linker_candidates*)
end
net = `build_net`(*MOF, node_candidates, linker_candidates*)

Algorithm 1: Fragmentation pseudocode. The `while` loop ensures that we do not classify metal-containing linkers (e.g., porphyrins) as metal clusters. Typically, only one — or in the case of porphyrin linkers — two loops are performed.

The `locate_unbound_solvent` function creates a $3 \times 3 \times 3$ supercell and analyzes if there are any non-periodic connected components. Those are floating molecules in the cell. In practice, we use a customized version of the `get_subgraphs_as_molecules` function in `pymatgen`.[29]

The `locate_nodes` function performs depth-first-search between all metals (ignoring those on the `ignored_metals` list) and potential branching sites. After clustering of neighboring branching sites and simplification in case multiple neighboring branching

sites are found, we identify the connected components that are spanned by the depth-first-search paths between metals and branching sites (where needed, we complete this path by also including, for example, bound hydrogen atoms on oxygen atoms connecting metals and branching sites). Those connected components are the metal nodes.

The `locate_bound_solvent` function checks for bridges on the node candidates. Note that, by default, we do not break those bridges. That is, the bound solvent remains bound to the nodes.

The `locate_linkers` function identifies the remaining connected components. For this, it deletes all solvent and node vertices (as well as the associated edges) from the structure graph. However, it keeps the branching indices. The remaining connected components are the linkers.

The `check_metal_in_linker` function attempts to identify linkers containing metals (e.g. porphyrins) that were incorrectly identified as metal clusters. For this, it checks for co-planarity of the metal and the branching sites.

We additionally ensure that the output molecules (in the linker and metal cluster collections) are correctly unwrapped by performing a breadth-first-search over the structure graph and picking the Cartesian coordinates of the neighbor image that is closest to the current Cartesian coordinates.

The `build_net` function uses the Cartesian coordinates of branching sites on metal clusters on linkers to identify connected building blocks and their barycenters. Additionally, we remove 2-connected vertices. To obtain an RCSR code, we input the labeled quotient graph in `CGD` format to the Systre program.[30]

All building blocks (metal clusters, linkers, solvent molecules) are stored in dedicated Python objects with wrapped molecules, original coordinates, and branching indices (among others) as attributes.

An example of the use of `moffragmentor` is given in Listing 1

```python
# load a CIF
mof = MOF.from_cif('tests/test_files/HKUST-1.cif')

# Fragment the MOF
fragments = mof.fragment()

# If you are in a Jupyter notebook you can visualize the components.
fragments.linkers[0].show_molecule()
fragments.nodes[0].show_molecule()


# You can also search PubChem for the building blocks
fragments.linkers[0].search_pubchem()


# To get the [RCSR code](http://rcsr.anu.edu.au/nets) run
fragments.net_embedding.rcsr_code
```

Listing 1 | Example for the use of the `moffragmentor`.

Supplementary Note 8 Leaderboard

Our website hosts multiple task-specific leaderboards. For each leaderboard, there is an interactive plot (comparing the metrics) along with a data table. The plots and data tables are automatically populated based on the `json` output of a `bench` run. In particular, we use custom Sphinx directives (via the `sphinxcontribs-needs` package) to standardize the metrics and implement interactive data tables with filtering functionality.

The pull request template we ask users to fill upon contributing a new model not only asks for the `json` file summarizing the metrics but also for a restructured text (`.rst`) file describing the model. This template also contains several questions inspired by the model cards proposed by Kapoor and Narayanan[31], intended to encourage submitters to reflect on potential data leakage.

References

1. Rose, M. E.; Kitchin, J. R. pybliometrics: Scriptable bibliometrics using a Python interface to Scopus. *SoftwareX* **2019**, *10*, 100263.

2. Moghadam, P. Z.; Li, A.; Wiggin, S. B.; Tao, A.; Maloney, A. G. P.; Wood, P. A.; Ward, S. C.; Fairen-Jimenez, D. Development of a Cambridge Structural Database Subset: A Collection of Metal–Organic Frameworks for Past, Present, and Future. *Chem. Mater.* **2017**, *29*, 2618–2625.

3. Manz, T. A.; Limas, N. G. Introducing DDEC6 atomic population analysis: part 1. Charge partitioning theory and methodology. *RSC Adv.* **2016**, *6*, 47771–47801.

4. Limas, N. G.; Manz, T. A. Introducing DDEC6 atomic population analysis: part 2. Computed results for a wide range of periodic and nonperiodic materials. *RSC Adv.* **2016**, *6*, 45727–45747.

5. Manz, T. A. Introducing DDEC6 atomic population analysis: part 3. Comprehensive method to compute bond orders. *RSC Adv.* **2017**, *7*, 45552–45581.

6. Limas, N. G.; Manz, T. A. Introducing DDEC6 atomic population analysis: part 4. Efficient parallel computation of net atomic charges, atomic spin moments, bond orders, and more. *RSC Adv.* **2018**, *8*, 2678–2707.

7. Rappe, A. K.; Casewit, C. J.; Colwell, K. S.; Goddard, W. A.; Skiff, W. M. UFF, a full periodic table force field for molecular mechanics and molecular dynamics simulations. *JACS* **1992**, *114*, 10024–10035.

8. Jablonka, K. M.; Ongari, D.; Smit, B. Applicability of Tail Corrections in the Molecular Simulations of Porous Materials. *J. Chem. Theory Comput.* **2019**, *15*, 5635–5641.

9. Willems, T. F.; Rycroft, C. H.; Kazi, M.; Meza, J. C.; Haranczyk, M. Algorithms and tools for high-throughput geometry-based analysis of crystalline porous materials. *Microporous Mesoporous Mater.* **2012**, *149*, 134–141.

10. Ongari, D.; Yakutovich, A. V.; Talirz, L.; Smit, B. Building a Consistent and Reproducible Database for Adsorption Evaluation in Covalent–Organic Frameworks. *ACS Cent. Sci.* **2019**, *5*, 1663–1675.

11. Dubbeldam, D.; Calero, S.; Ellis, D. E.; Snurr, R. Q. RASPA: molecular simulation software for adsorption and diffusion in flexible nanoporous materials. *Mol. Simulat.* **2015**, *42*, 81–101.

12. Potoff, J. J.; Siepmann, J. I. Vapor–liquid equilibria of mixtures containing alkanes, carbon dioxide, and nitrogen. *Aiche J.* **2001**, *47*, 1676–1682.

13. Bucior, B. J.; Bobbitt, N. S.; Islamoglu, T.; Goswami, S.; Gopalan, A.; Yildirim, T.; Farha, O. K.; Bagheri, N.; Snurr, R. Q. Energy-based descriptors to rapidly predict hydrogen storage in metal–organic frameworks. *Mol. Syst. Des. Eng.* **2019**, *4*, 162–174.

14. Michels, A.; de Graaff, W.; Ten Seldam, C. A. Virial coefficients of hydrogen and deuterium at temperatures between −175°C and +150°C. Conclusions from the second virial coefficient with regards to the intermolecular potential. *Physica* **1960**, *26*, 393–408.

15. Darkrim, F.; Levesque, D. Monte Carlo simulations of hydrogen adsorption in single-walled carbon nanotubes. *J. Chem. Phys.* **1998**, *109*, 4981–4984.

16. Martin, M. G.; Siepmann, J. I. Transferable potentials for phase equilibria. 1. United-atom description of n-alkanes. *The Journal of Physical Chemistry B* **1998**, *102*, 2569–2577.

17. Moghadam, P. Z.; Islamoglu, T.; Goswami, S.; Exley, J.; Fantham, M.; Kaminski, C. F.; Snurr, R. Q.; Farha, O. K.; Fairen-Jimenez, D. Computer-aided discovery of a metal-organic framework with superior oxygen uptake. *Nat. Commun.* **2018**, *9*, 1378.

18. Zhang, L.; Siepmann, J. I. Direct calculation of Henry's law constants from Gibbs ensemble Monte Carlo simulations: nitrogen, oxygen, carbon dioxide and methane in ethanol. *Theor. Chem. Acc.* **2006**, *115*, 391–397.

19. Boato, G.; Casanova, G. A self-consistent set of molecular parameters for neon, argon, krypton and xenon. *Physica* **1961**, *27*, 571–589.

20. Abascal, J. L.; Vega, C. A general purpose model for the condensed phases of water: TIP4P/2005. *J Chem Phys* **2005**, *123*, 234505.

21. Cho, E. H.; Lin, L.-C. Electrostatic potential optimized molecular models for molecular simulations: CO, CO_2, COS, H_2S, N_2, N_2O, and SO_2. *J. Chem. Theory Comput.* **2019**, *15*, 6323–6332.

22. Huck, J. M.; Lin, L.-C.; Berger, A. H.; Shahrak, M. N.; Martin, R. L.; Bhown, A. S.; Haranczyk, M.; Reuter, K.; Smit, B. Evaluating different classes of porous materials for carbon capture. *Energy Environ. Sci.* **2014**, *7*, 4132–4146.

23. Moosavi, S. M.; Novotny, B. Á.; Ongari, D.; Moubarak, E.; Asgari, M.; Özge Kadioglu,; Charalambous, C.; Ortega-Guerrero, A.; Farmahini, A. H.; Sarkisov, L.; Garcia, S.; Noé, F.; Smit, B. A data-science approach to predict the heat capacity of nanoporous materials. *Nat. Mater.* **2022**, *21*, 1419–1425.

24. Le, T. T.; Fu, W.; Moore, J. H. Scaling tree-based automated machine learning to biomedical big data with a feature set selector. 2020.

25. Hedges, L. V. Distribution Theory for Glass's Estimator of Effect size and Related Estimators. *J. Educ. Stat.* **1981**, *6*, 107–128.

26. Cumming, G. *Understanding the new statistics: Effect sizes, confidence intervals, and meta-analysis*; Routledge, 2013.

27. Sawilowsky, S. S. New Effect Size Rules of Thumb. *Journal of Modern Applied Statistical Methods* **2009**, *8*, 597–599.

28. Shervashidze, N.; Schweitzer, P.; Van Leeuwen, E. J.; Mehlhorn, K.; Borgwardt, K. M. Weisfeiler-lehman graph kernels. *J. Mach. Learn. Res.* **2011**, *12*.

29. Ong, S. P.; Richards, W. D.; Jain, A.; Hautier, G.; Kocher, M.; Cholia, S.; Gunter, D.; Chevrier, V. L.; Persson, K. A.; Ceder, G. Python Materials Genomics (pymatgen): A robust, open-source python library for materials analysis. *Comput. Mater. Sci.* **2013**, *68*, 314–319.

30. Delgado-Friedrichs, O.; O'Keeffe, M. Identification of and symmetry computation for crystal nets. *Acta Cryst Sect A* **2003**, *59*, 351–360.

31. Kapoor, S.; Narayanan, A. Leakage and the Reproducibility Crisis in ML-based Science. *arXiv preprint arXiv: Arxiv-2207.07048* **2022**,
