# Supplementary material for: An Ecosystem for Digital Reticular Chemistry
Source: ACS Cent Sci. 2023 Mar 10;9(4):563–81. doi: 10.1021/acscentsci.2c01177 (PMC10141625; doi:10.1021/acscentsci.2c01177)
Supplement: Supplementary file 2 — oc2c01177_si_002.pdf [file oc2c01177_si_002.pdf]

RESPONSE TO THE REVIEWERS

REVIEWER 1

In this work, the authors present a Python package, mofdscribe, that provides utilities reticular chemistry along each step along the path from ideation to model publication. They optimized the software as a universal tool to make machine learning for reticular chemistry a science instead of an art. Their ecosystem provides machine learning-ready datasets, along with more than 35 reported and unreported featurization approaches, under a consistent application programming interface (API) that enables rapid experimentation and makes those tools accessible to non-experts. In addition, data splitters, as well as benchmarking tools are provided to embrace a community effort in which bugs are fixed and new features are added by the community of digital chemists and materials scientist.

Reviewer Point P 1.1 — Whilst I think this has potential as a research area it does not really present a step forward yet. What is missing is an ontology that allows the instructions to be instantiated in the physical world. For this to be acceptable for in focus the authors need to make a bridge to users who will be designing and then making new reticular materials.

Reply: *Linking computational research with experimental work is of paramount importance. To highlight the role our ecosystem can play in this, we added*

> *This will allow for a closer coupling of data-driven materials design and the synthesis and characterization of (in silico generated) materials since it is very easy for non-experts to use mofdscribe to power machine-learning models that could be used, for instance, in an active learning workflow.*[1]

to the main text. We also added a reference to Bai et al.[2] *to underline the need for machine-actionable data for full integration in self-driving labs. Additionally, we want to emphasize that we started some efforts to make datasets more semantic (see* `https://github.com/kjappelbaum/mofdscribe/issues/345`*)*

Additionally, we now provide an interface for the thermal stability dataset reported by[3] *and also use it for an interactive demo of mofdscribe, which users can run without installation on Google Colab to experience how mofdscribe can also be used on experimental data. We added a reference to this example in the caption of the case study "Creating a new model and submitting it to the leaderboard":*

> *More examples, including one on an experimental dataset, can be found in in the GitHub repository (*`https://github.com/kjappelbaum/mofdscribe/tree/main/examples`*). The examples can be run on Google Colab (e.g.* `https://colab.research.google.com/github/kjappelbaum/mofdscribe/blob/main/examples/build_model_using_mofdscribe.ipynb`*.*

To illustrate that machine learning for reticular chemistry and porous materials is already widely used, we also added Supplementary Figure 1, which shows the growth of the number of publications per year in this field. Given the growth of the field, we find it of paramount importance to propose some standard framework that helps researchers to stick to best practices, while also making their works more comparable.

REVIEWER 2

The authors present mofdscribe which is a Python library for digital reticular chemistry. The tool contains multiple features that have been adapted from publications. The featurization is easy to use and there is lots of useful/online information on installation, documentation, featurization, machine learning algorithms, datasets etc: see https://mofdscribe.readthedocs.io/en/latest/index.html These tools are probably more interesting for less experienced users to take advantage of machine learning in the domain of digital chemistry

Reviewer Point P 2.1 — For the wide range of applications that MOFs are being studied for, I think there is room for improvement to include more descriptors on surface chemistry, special adsorbate-MOF interactions, gas mixture predictions, water affinity and many others. This would be outside the scope of the current work, but I am sure that over time, with the aid of the MOF community, this interesting model prediction software will be even more useful in designing and discovering ideal candidates for different MOF applications.

Reply: *To emphasize that mofdscribe can be easily coupled with matminer, which already implements tools for surface chemistry, we added*

> *For instance, featurizers such as the `matminer`'s `SiteStatsFingerprint` can be seamlessly used to separately featurize framework and guest molecules using the `HostGuestFeaturizer` implemented in `mofdscribe`.*

to the main text.

Additionally, we also implemented a wrapper featurizer class `HostGuestFeaturizer` (similar to the `BUFeaturizer` that computes separately for the MOF building blocks) that computes features (e.g., SOAP, RACs, . . .) separately for the framework and guest molecules. In addition, we also implement a guest-centered version of the atom-property-labeled autocorrelations functions (which, to our knowledge, has not been reported so far). We provide more details on the use of those new featurizers in the documentation (`https://mofdscribe.readthedocs.io/en/latest/background.html#host-guest-featurization`)

This is an excellent contribution and I suggest it for publication.

REFERENCES

[1] Jablonka, K. M.; Jothiappan, G. M.; Wang, S.; Smit, B.; Yoo, B. *Nat. Commun.* **2021**, *12*.

[2] Bai, J.; Cao, L.; Mosbach, S.; Akroyd, J.; Lapkin, A. A.; Kraft, M. *JACS Au* **2022**, *2*, 292–309.

[3] Nandy, A.; Terrones, G.; Arunachalam, N.; Duan, C.; Kastner, D. W.; Kulik, H. J. *Sci. Data* **2022**, *9*.

RESPONSE TO THE REVIEWERS

REVIEWER 1

Reviewer Point P 1.1 — My comments are addressed by the authors.

REVIEWER 2

Reviewer Point P 2.1 — The manuscript by Smit, Berend oc-2022-01177p.R1 18-Jan-202 is important and I think the impact will be increased if they can describe a roadmap to connect their digital workflow to a practically executable workflow – i.e. what was needed on the adoption side, concrete abstraction (c.f. XDL for the chemputer) and potential hardware modules. With these reflections I think the impact of the article will be increased since it will inspire researchers to set up both digital and physical workflows.

Reply: *We fully agree that this is an important point. In our original discussion, we had a much more limited focus on making sure that with a given data set, we draw the right conclusions from an ML study. However, the reviewer makes a good point that an ML study does not stop there, and we added that the proper ML conclusions allow us to tackle the next challenge:*

> *For this, the adoption of suitable physical workflows will be beneficial. Via tools such as the χDL[1] and the ChemPU[2] predictions enabled by mofdscribe could guide an autonomous laboratory if the predictions can be mapped to instructions that can be executed on laboratory robots.[3,4]*

REFERENCES

[1] Mehr, S. H. M.; Craven, M.; Leonov, A. I.; Keenan, G.; Cronin, L. *Science* **2020**, *370*, 101–108.

[2] Steiner, S.; Wolf, J.; Glatzel, S.; Andreou, A.; Granda, J. M.; Keenan, G.; Hinkley, T.; Aragon-Camarasa, G.; Kitson, P. J.; Angelone, D.; Cronin, L. *Science* **2019**, *363*.

[3] Wilbraham, L.; Mehr, S. H. M.; Cronin, L. *Acc. Chem. Res.* **2020**, *54*, 253–262.

[4] Hammer, A. J. S.; Leonov, A. I.; Bell, N. L.; Cronin, L. *JACS Au* **2021**, *1*, 1572–1587.
